# B591, a novel specific pan-PI3K inhibitor, preferentially targets cancer stem cells

**DOI:** 10.1038/s41388-018-0674-5

**Published:** 2019-01-11

**Authors:** Hongyu Zhou, Chunlei Yu, Lingmei Kong, Xiaoliang Xu, Juming Yan, Yingchao Li, Tao An, Liang Gong, Yaxiao Gong, Huifang Zhu, Hongbin Zhang, Xiaodong Yang, Yan Li

**Affiliations:** 1 State Key Laboratory of Phytochemistry and Plant Resources in West China, Kunming Institute of Botany, Chinese Academy of Sciences, Kunming, China; 2 Institute of Materia Medica, School of Pharmacy, North Sichuan Medical College, Nanchong, China; 3 Key Laboratory of Medicinal Chemistry for Natural Resource, Ministry of Education and Yunnan Province, School of Chemical Science and Technology, Yunnan University, Kunming, China; 4 University of the Chinese Academy of Sciences, Beijing, China; 5 Yunnan Key Laboratory of Natural Medicinal Chemistry, Kunming Institute of Botany, Chinese Academy of Sciences, Kunming, China

**Keywords:** Target validation, Cancer stem cells, Growth factor signalling

## Abstract

Cancer stem cells (CSCs) have been implicated in metastasis, relapse, and therapeutic resistance of cancer, so successful cancer therapy may therefore require the development of drugs against CSCs or combining anti-CSCs drugs with conventional therapies. The phosphoinositide 3-kinase (PI3K) signaling pathway is one of the most frequently activated signaling pathways in human cancer, playing a central role in tumorigenesis as well as the maintenance of CSCs. Here, we designed and identified B591, a dihydrobenzofuran-imidazolium salt, as a novel specific pan-PI3K inhibitor with potent inhibitory activity against class I PI3K isoforms, which showed effective inhibition of cellular PI3K/mTOR signaling pathway and robust antitumor activity in a set of cancer cell lines. Notably, compared with bulk tumor cell populations, B591 exhibited more potency in suppressing CSCs survival and inducing CSCs apoptosis, and presence of B591 effectively eliminated paclitaxel-enriched CSCs. B591 diminished self-renewal capacity and decreased the expression of epithelial-mesenchymal transition (EMT) markers of CSCs. In vivo, B591 preferentially decreased CSCs levels in mouse xenograft model of human breast cancer as evidenced especially by remarkable reduction of tumor-initiating ability. Consistent with the preferential targeting of CSCs, B591 effectively inhibited breast tumor metastasis and delayed tumor regrowth following paclitaxel treatment. Taken together, our findings establish B591, a novel PI3K inhibitor, as a strong candidate for clinical evaluation as a CSCs targeting agent.

## Introduction

Tumor relapse and metastasis remain major obstacles in the improvement of overall cancer survival. A small subpopulation of cancer cells termed cancer stem cells (CSCs) have been proposed as the driving force of tumorigenesis and the seeds of metastases [1]. Since firstly identified in human acute myeloid leukemia, CSCs have been discovered in many other solid tumor types including breast cancer [2–5]. Featured with capacity of tumor-initiation and self-renewal, CSCs are implicated in tumorigenesis, metastasis, relapse, chemo-, and radio-resistance of cancers [1]. Therefore, successful cancer therapy may require the development of drugs targeting CSCs or combining anti-CSCs drugs with conventional therapies [6].

Phosphatidylinositol 3-Kinases (PI3Ks) are a family of lipid kinases that play an important role in regulating various physiological functions and cellular processes [7, 8]. Dysregulation of the PI3K signaling pathway has been implicated in a wide range of tumor types as a result of genetic and epigenetic aberrations [9]. These aberrations lead to increased downstream signaling through kinases including Akt and mTOR [10]. mTOR functions as two distinct signaling complexes, mTOR complex 1 (mTORC1) and mTORC2, each with different downstream substrates [7, 8, 10]. Tremendous efforts have been dedicated to discover and develop effective anticancer drug targeting PI3K and/or mTOR, and several pan-specific, isoform-specific PI3K inhibitors or PI3K/mTOR dual inhibitors have been identified in the past decade. However, due to earlier problems, such as unfavorable pharmacokinetic profiles and high in vivo toxicity, there is a continually growing need to discover novel PI3K and/or mTOR inhibitors for further development into therapeutic candidates for cancer treatment [11].

Accumulating evidence has shown that PI3K/mTOR signaling pathway plays a critical role in CSCs [12, 13]. In breast cancer, the activation of mTOR is required for CSCs maintenance and viability, and activation of PI3K/Akt, achieved by knocking down PTEN, enriched breast CSCs [14, 15]. In prostate cancer cells, PI3K subunits p110α and p110β-protein levels were higher in cells grown under sphere-forming conditions, and stable knockdown of PTEN by shRNA resulted in an increased sphere-forming ability as well as increased clonogenic and tumorigenic potential. Meanwhile, inhibition of the PI3K pathway by PI3K inhibitor LY290042 led to a decrease in CD133^+^/CD44^+^ stem-like populations in prostate cancer cell lines [12]. These studies strongly suggest that the PI3K/mTOR pathway is critical for CSCs maintenance and that targeting PI3K signaling may be beneficial in cancer treatment through eliminating CSCs.

**Fig. 1:**
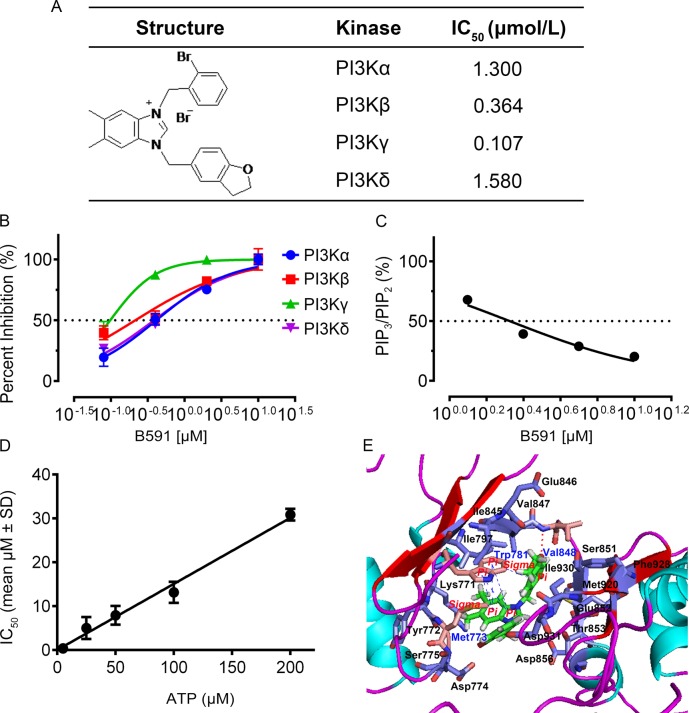
B591 is a selective ATP-competitive inhibitor of Class I PI3K kinase. **a** The chemical structure of B591 and IC_50_ values of B591 for PI3K α, β, γ, and δ isoforms. **b** In vitro PI3K kinase assay. Purified PI3K Class I Lipid Kinase was respectively incubated with B591 for 30 min at room temperature. The activity of PI3K was evaluated using a PI3-Kinase Activity ELISA kit according to the manufacture’s instructions. **c** Phospholipids were isolated from HCT116 cells treated with DMSO or B591 for 10 h and relative PIP_3_ and PI(4,5)P_2_ levels were quantified by ELISA. For normalization, the PIP_3_/PI(4,5)P_2_ ratio was set to 100% for DMSO treated cells. **d** IC_50_ values of B591 are plotted against the concentration of ATP in the PI3Kβ enzymatic assay. **e** Proposed binding mode of B591 with PI3Kβ

In the present study, we designed and synthesized a series of 5-subsitituted dihydrobenzofuran-imidazolium salts compounds and identified a novel specific pan-PI3K inhibitor B591, 3-(2-bromobenzyl)-1-((2,3-dihydrobenzofuran-5-yl)methyl) -5,6-dimethyl-1*H*-benzo[*d*]imidazol-3-ium bromide (Fig. 1a), which exhibited approximately equal potency against Class I isoforms of PI3K, while little inhibitory activity was observed in 39 other closely related protein and lipid kinases, indicating the high selectivity towards PI3K. In cellular assays, B591 potently inhibited the dysfunctional activation of PI3K/mTOR pathway and attenuated the growth of a panel of cancer cell lines. Notably, B591 preferentially targeted and exhibited profound effects on CSCs subpopulation, as demonstrated by CSCs phenotype assay and the limiting dilution tumor-initiating assay. Consistent with the preferential targeting of CSCs, B591 effectively inhibited tumor metastasis and delayed the relapse of breast cancer following cessation of paclitaxel treatment in human breast cancer xenografted models.

## Results

### B591 is a selective ATP-competitive inhibitor of Class I PI3K kinase

A series of 5-subsitituted dihydrobenzofuran-imidazolium salts compounds were synthesized as described (Supplementary Fig. S1-S4). The biochemical potency of these compounds against the class I PI3K isoforms was determined with PI3-Kinase Activity ELISA assay, and B591 (Fig. 1a) was identified as a potent pan-PI3K inhibitor. As shown in Fig. 1a, b, B591 potently inhibited the kinase activity of the four Class I PI3Ks isoforms (IC_50_: PI3Kα = 1.300 ± 0.27 μmol/L; PI3Kβ = 0.364 ± 0.13 μmol/L; PI3Kγ = 0.107 ± 0.07 μmol/L; PI3Kδ = 1.580 ± 0.16 μmol/L). To further confirm the inhibitory activity of B591 on Class I PI3Ks, PIP_3_ Mass ELISA which directly detect and quantify PI(3,4,5)P_3_ level from cells were used. In HCT116 cells, PIP_3_/PI(4,5)P_2_ ratios were measured after treatment with various concentrations of B591. As shown in Fig. 1c, B591 treatment induced a dose-dependent decrease of PIP_3_/PI(4,5)P_2_ ratio (IC_50_ = 1.910 μmol/L), suggesting that B591 is able to inhibit PI3K kinase activity at cellular level effectively. Furthermore, the kinase selectivity spectrum of B591 was tested against a broad panel of other protein and lipid kinases. B591 showed a highly selective profile when tested against additional 39 human protein kinases, including PIKK family members and PI3K pathway involved kinases (Supplementary Table S1; Life technologies). Only PI3K-C2α, hVPS34, and PI3K-C2β were inhibited by B591 at IC_50_ values of ~100 μmol/L.

Most of the PI3K inhibitors currently available are reversibly ATP-competitive. At a certain concentration of ATP, stoichiometric amount of inhibitor is required to displace the ATP-binding pocket of PI3K, so the increase of ATP concentration contributes to the decreased activity of tested compound in PI3K kinase inhibition. The IC_50_ value for inhibition of PI3Kβ by B591 was further determined in the presence of various concentrations of ATP (5 μM, 25 μM, 50 μM, 100 μM, and 200 μM). The analysis showed that increasing concentrations of ATP reduced the inhibitory efficacy of B591 against PI3Kβ (Fig. 1d), indicating that B591 is an ATP-competitive inhibitor of PI3K kinase. The PI3K active site contains three key regions: the hinge region, the affinity pocket and the ribose pocket, of which the hinge region binder is crucial for PI3K inhibitors and the affinity pocket interaction contributes to improved potency and potential selectivity [16]. Analysis of B591 docked into p110β (PDB:2Y3A) showed that the oxygen atom in 2,3-dihydrobenzofuran-ring of B591 formed critical hydrogen bonds with Val848 in the hinge region, while the benzimidazole ring interacted with Met773 and Trp781 in the ribose pocket (Fig. 1e). This molecular docking model indicated that B591 is able to bind with the ATP-binding pocket of PI3K, further demonstrating that B591 is an ATP-competitive inhibitor of class I PI3K.

Furthermore, Molinspiration server and SwissADME server was respectively used to quickly understand the physicochemical and ADME properties of B591 (Supplementary Table. S2a and S2b). These analyses suggest that B591 possesses a generally favorable ADME profile and could be a potential lead compound for further development.

### B591 inhibits PI3K/Akt pathway in multiple cancer cell lines

**Fig. 2:**
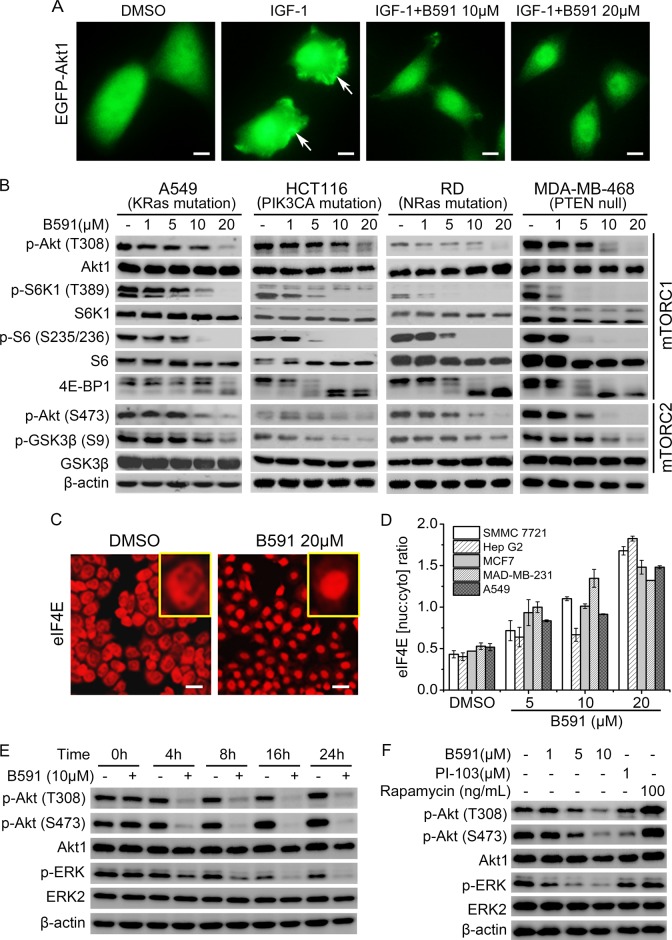
B591 inhibits PI3K/Akt pathway in multiple cancer cell lines. **a** Representative images of Akt1 redistribution in IGF-1-treated cells in the absence or presence of B591. Scale bar represents 10 μm. Arrows indicate IGF-1-mediated membrane translocation detected by the image analysis algorithm. **b** Inhibition of mTORC1 and mTORC2 signaling in multiple cancer cell lines, exposed for 5 h to increasing concentrations of B591. **c** HepG2 cells were treated with DMSO or 20 μM of B591 for 5 h. Immunofluorescence (red) intensity reflects eIF4E subcellular distribution. Cells were visualized with high content reader. Representative images are shown, and scale bar represents 50 μm. **d** Nuclear-to-cytoplasmic ratio of eIF4E in five cancer cell lines was determined via high content reader. **e** The phosphorylation of AKT and ERK was determined by immunoblotting in RD cells in the presence of 10 μM of B591 after indicated time of exposure. **f** Inhibition of the phosphorylation of AKT and ERK in RD cells treated with B591, PI-103 or Rapamycin for 24 h was determined by immunoblotting

Growth factors such as insulin-like growth factor 1 (IGF-1) and cytokines such as interleukin-6 stimulate and activate PI3K, leading to a subsequent increase of plasma membrane translocation and phosphorylation of Akt. Activated Akt promotes cell survival, cell growth and proliferation in human cancers [17]. Using ectopic expressed Akt-EGFP fusion protein as a probe, we showed that IGF-1 significantly stimulated translocation of Akt to the plasma membrane in CHO cells stably expressing EGFP-Akt1, whereas, B591 treatment potently prevented this translocation event in a concentration-dependent manner (Fig. 2a).

The key effectors downstream of PI3K were examined by western blot analysis in multiple tumor cell lines with different genetic background [18–20]. As shown in Fig. 2b, B591 potently inhibited the phosphorylation of Akt at Thr308, a direct substrate of PDK1 and an indirect substrate of PI3Ks (Fig. 2b). Subsequently, the phosphorylation of 4E-BP1, S6K1 and its substrate ribosomal S6 were also suppressed by B591 treatment, indicating that B591 effectively inhibited PI3K/Akt/mTORC1 signaling. Most recent study identified PtdIns(3,4,5)*P*_3_, which is generated by activated PI3K, as a direct upstream activator for mTORC2, highlighting the critical role of PI3K in mTORC2 activation [21]. Therefore, the effect of B591 on mTORC2 signaling was evaluated by testing the phosphorylation levels of Akt at Ser473 and its substrate GSK3β at Ser9. In all tested cell types, B591 induced a concentration-dependent decrease of p-Akt (Ser473) and p-GSK3β (Ser9) (Fig. 2b). As a positive control, PI-103, which is a dual PI3K/mTOR inhibitor, inhibited PI3K downstream mTORC1 and mTORC2 signaling (Supplementary Fig. S5A). In contrast, a commonly used chemotherapy-drug paclitaxel, did not show any effect on PI3K/Akt signaling (Supplementary Fig. S5A). Moreover, B591 also inhibited the activation of mTORC1 and mTORC2 signaling caused by PI3K activation after IGF-1 stimulation (Supplementary Fig. S5B).

The mRNA 5′ cap-binding protein eukaryotic initiation factor 4E (eIF4E) is a key regulator of cap-dependent translation initiation in the cytoplasm. Elevated level of eIF4E has been implicated in oncogenic transformation and tumorigenesis [22]. The availability of eIF4E is tightly controlled through regulated interaction with 4E-BPs [22, 23]. eIF4E is predominantly cytoplasmic in mammalian cells, whereas serum-starvation and/or rapamycin-treatment: conditions that suppress mTOR signaling induces an accumulation of eIF4E within the nucleus [24]. By using immunofluorescence assay, we assessed the effect of B591 on eIF4E subcellular localization of eIF4E. As expected, eIF4E in untreated HepG2 cells was dominantly localized in cell cytoplasm, treatment of the cells with 20 μM of B591 obviously induced the nuclear translocation of eIF4E (Fig. 2c). Further, five human cancer cell lines (SMMC7721, HepG2, MCF7, MDA-MB-231, and A549) were respectively treated with different concentrations of B591 for 5 h and eIF4E [nuc:cyto] ratios were determined by a high content assay using Thermo Scientific Cellomics ArrayScan VTI HCS. The results showed that B591 increased eIF4E [nuc:cyto] ratios in all the five cancer cells in a dose-dependent manner (Fig. 2d).

### B591 prevents feedback activation of Akt and ERK

Despite a significant efficacy in preclinical models, the clinical tumor response to mTORC1 inhibitors, such as rapalogs, is modest [25]. The presence of different feedback loops resulting in a hyper-activation of Akt and MAPK, thus promoting cell proliferation and survival, is one of the factors that limited the clinical application of mTORC1 inhibitors [26–28]. To gain insights into the activity of Akt and ERK after PI3K/mTOR signaling inhibition by B591, we further analyzed the time-course phosphorylation of Akt and ERK after incubation of rhabdomyosarcoma RD cells and human breast cancer MDA-MB-231 cells with B591. As shown in Fig. 2e, 10 μM of B591 treatment effectively suppressed the phosphorylation of Akt and ERK over the whole time course of 24 h in RD cells. In contrast, mTOR inhibitor rapamycin induced a hyper-activation of Akt and increased ERK phosphorylation after 24 h treatment (Fig. 2f), consistent with the release of the S6K-IRS1-Akt and S6K-PI3K-MAPK feedback loops previously reported [27, 28]. Compared with PI-103, a PI3K/mTOR dual inhibitor, B591 more effectively inhibited the phosphorylation of both Akt and ERK (Fig. 2f). Similar results were obtained in MDA-MB-231 cells (Supplementary Fig. S6). With the capacity of effectively preventing feedback activation of both Akt and ERK, B591 may achieve a robust anticancer effect in clinical application.

### B591 inhibits cancer cell proliferation and induces apoptosis

The anti-proliferative potency of B591 was examined in a panel of cells derived from different cancer types with different mutation status (Supplementary Fig. S7A and S7B). Cyclin D1 and p27 which are two downstream effectors of PI3K/Akt signaling perform important functions in the control of cell cycle progression [29]. We assumed a cell cycle arrest upon PI3K inhibition by B591. Cell cycle analysis in MDA-MB-231 cells showed that B591 effectively arrested cells in G0/G1 phase, which was accompanied with a concomitant decrease of cell number in S phase and G2/M phase of the cell cycle (Supplementary Fig. S8A). Consistently, B591 strongly decreased the protein expression of cyclin D1 and increased the expression of p27 (Supplementary Fig. S8B). Apoptosis induced by B591 was further investigated and the results showed that B591 exhibited a significant apoptosis inducing effect (Supplementary Fig. S8C and S8D), which is associated with the enhanced cleavage of PARP and caspase-3 and the decreased expression of Bcl-XL (Supplementary Fig. S8E).

### B591 preferentially targets CSCs

The inhibitory activity of B591 on CSCs and bulk tumor cells was investigated and the results showed that B591 preferentially suppressed CSCs, with paclitaxel, a conventional chemotherapeutic drug, as a comparison. Breast cancer cell population with CD44^high^/CD24^low^ phenotype or high aldehyde dehydrogenase (ALDH) activity has been shown to enrich breast CSCs [30]. In our study, mammospheres with the characteristics of stem-like cells were collected by long-term serum-free suspension cultivation (Supplementary Fig. S9A) from MDA-MB-231 and SUM-159PT breast cancer cells, both of which possess high stemness and are commonly used in breast cancer stem cell study [31, 32]. As shown in Supplementary Fig. S9B, the percentage of ALDH^high^ cells were dramatically increased in MDA-MB-231 and SUM-159PT derived mammospheres. Meanwhile, higher expression level of CSC markers, such as ALDH, CD44, BMI1, SOX2, and Nanog, were observed in sphere-forming cells than in monolayer cells (Supplementary Fig. S9C). Moreover, the mammosphere cells showed higher activity in PI3K/mTOR pathway as featured by the increased phosphorylation status of its downstream substrates S6K1 and S6 (Supplementary Fig. S9D). When incubated into the mammary fat pad of NOD/SCID mice (1000 cells), the mammosphere cells (3 out of 4) exhibited more tumorigenic potential than the monolayer cells (0 out of 4) (Supplementary Fig. S9E).

**Fig. 3:**
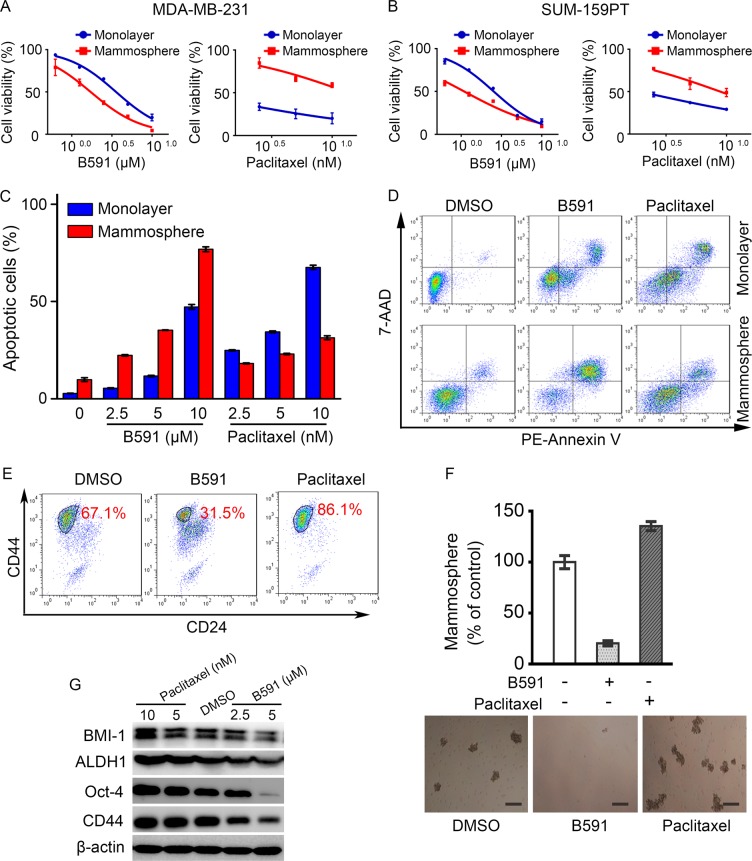
B591 preferentially targets CSCs. **a**, **b** B591 preferentially reduced the viability of mammosphere of MDA-MB-231 and SUM-159PT cell lines. Inhibitory activity of B591 and paclitaxel on the proliferation of monolayer and mammosphere cells was analyzed, and data represent mean ± SD. **c** B591 preferentially induced the apoptosis of mammosphere cells of MDA-MB-231 cell line. Cells were treated with indicated agents for 48 h, and cells were harvested and processed for apoptosis assay using the PE-Annexin V/7-AAD Apoptosis Detection Kit. **d** Representative flow cytometry plots of apoptosis inducing activity of B591 (10 μM) and paclitaxel (10 nM) on monolayer and mammosphere MDA-MB-231 cells. **e** B591 preferentially targeted CD44^high^/CD24^low^ cells. Cells were treated with B591 or paclitaxel for 2 days. Following a 4-day recovery period, cells were co-stained with CD44 and CD24 antibodies and subjected to FACS analysis. Representative flow cytometry plots show the effect of B591 on CD44^high/^CD24^low^ population in MDA-MB-231 cells in vitro. **f** B591 decreased mammosphere forming efficiency. Primary MDA-MB-231 mammospheres were treated with B591 or paclitaxel for 48 h. Results of the secondary mammosphere-formation assay are shown. Scale bar represents 40 μm. **g** B591 decreased the expression of CSC markers in MDA-MB-231 cells. Cells were treated with B591 or paclitaxel for 2 days. Following a 4-day recovery period, the expression of CSC markers was analyzed with western blot assay

The proliferation inhibitory activities of B591 were compared between monolayer and mammosphere cells. As shown in Fig. 3a, b, although B591 dose-dependently inhibited both monolayer and mammosphere cell populations, it was more potent against mammosphere cells from MDA-MB-231 and SUM-159PT respectively, whereas mammosphere cells were resistant to the conventional therapeutic agent paclitaxel. Consistently, in both MDA-MB-231 (Fig. 3c, d) and SUM-159PT (Supplementary Fig. S10A and S10B) cells, B591 preferentially induced the apoptosis of mammosphere populations, while paclitaxel induced less apoptosis of mammosphere populations than monolayer populations.

In breast cancer, AI-Hajj et al. firstly identified and isolated CSCs based on the expression status of the specific cell surface markers CD44 and CD24. The population with CD44^+^/CD24^−/low^ phenotype had significantly higher tumorigenic potential when injected into the mammary fat pad of female NOD/SCID mice than CD44^+/−^/CD24^+^ cell fractions [3]. Our study showed that the percentage of CD44^high^/CD24^low^ cells was significantly increased in paclitaxel treated breast cancer cells, indicating the proportion of CSCs in the tumor was enriched by paclitaxel, consistent with the reports that CSCs are resistant to the conventional therapies, and these therapies rather enrich the proportion of CSCs in the tumor by eliminating non-stem tumor bulk cells [33, 34]. Strikingly, B591 induced distinct decrease of CD44^high^/CD24^low^ population in both MDA-MB-231 (Fig. 3e) and SUM-159PT (Supplementary Fig. S10C) cells.

In the mammosphere assay, MDA-MB-231 (Fig. 3f) and SUM-159PT (Supplementary Fig. S10D) cells were treated with B591 or paclitaxel, and the mammospheres were collected and subsequently passaged. Secondary Mammosphere-forming activity was determined, which reflects the self-renewal capability of CSCs [35]. The results showed that the mammosphere-forming ability was strikingly suppressed by B591 but increased by paclitaxel. Western blot assay further demonstrated that B591 dramatically decreased the expression of CSC markers, including BMI-1, ALDH1, Oct-4, and CD44 in both MDA-MB-231 (Fig. 3g) and SUM-159PT (Supplementary Fig. S10E) cells.

### B591 reduces breast CSCs and inhibits tumorigenesis

**Fig. 4:**
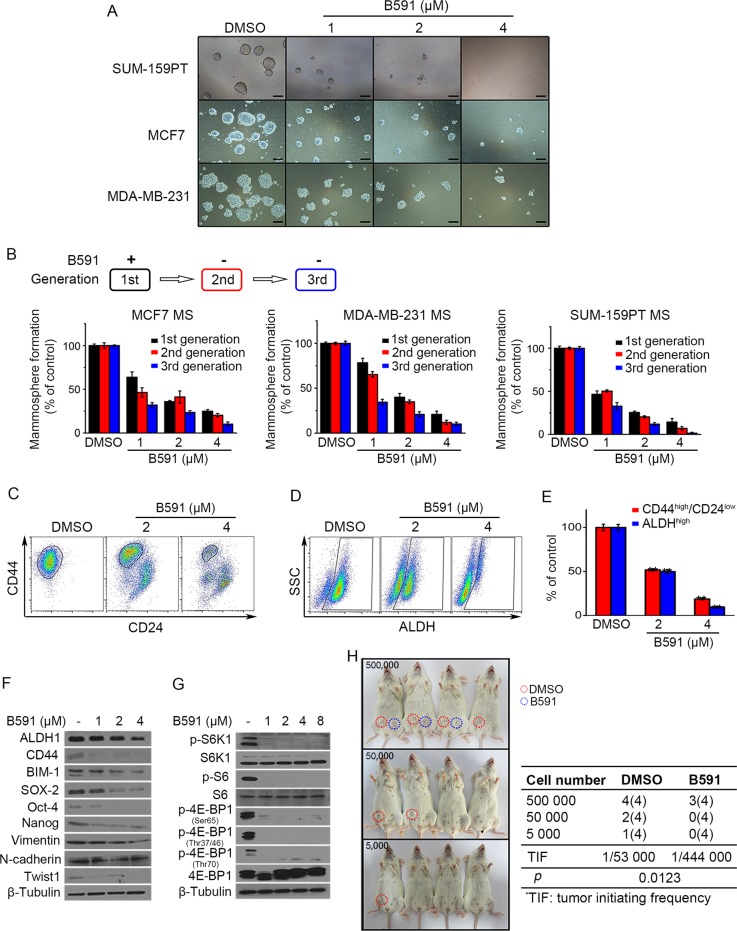
B591 inhibits CSCs and tumorigenesis. **a** B591 inhibited mammosphere-formation of SUM-159PT, MCF7, and MDA-MB-231 cells. Mammospheres were cultured as described in Materials and methods section. In mammosphere formation assay, mammospheres were treated with indicated concentrations of B591 for 5 days and imaged under a phase-contrast microscopy. Representative images are shown, and scale bar represents 40 μm. **b** The 1st generation mammospheres were treated with indicated concentrations of B591 for 5 days and counted under a phase-contrast microscopy. The mammospheres were subsequently passaged for another two generations and each generation of the mammospheres were cultured for 5 days without B591 and then counted under a phase-contrast microscopy. The relative mammosphere-formation ability is shown as percentage, and data represent mean ± SD from three independent experiments. **c**, **d** Representative flow cytometry plots show the effect of B591 on CD44^high^/CD24^low^ (**c**) and ALDH^high^ (**d**) breast CSCs population from SUM-159PT cells. **e** Quantification of flow cytometry analysis of CD44^high^/CD24^low^ and ALDH^high^ breast CSCs population from SUM-159PT cells. Data from two independent experiments are plotted. ^**^p < 0.01, difference versus DMSO-treated control group. **f**, **g** A panel of breast CSCs marker and the downstream substrates of PI3K pathway were examined by western blot analysis using lysate from SUM-159PT mammospheres. **h** Limiting dilution assay shows the effect of B591 (4 μM) on tumor-initiating ability of SUM-159PT cells. SUM-159PT cells were pretreated with 4 μM of B591 for 24 h and then cultured for 48 h without B591. The live cells were collected and counted, the same amount of control cells and B591-treated cells were then incubated into the fourth left and right mammary fat pad of mice, respectively, according the indicated number for each group. Tumors were inspected 3 months later, and the tumor initiating frequency (TIF) was calculated with ELDA software. Numbers in the column DMSO and B591 are illustrated as: number of tumor-bearing mice (number of recipient mice)

The observation that B591 preferentially targets CSCs promoted us to further evaluate the effect of B591 in multiple orthogonal CSC assays. Firstly, mammosphere formation assay was performed in breast cancer cell lines SUM-159PT, MCF7, and MDA-MB-231. Mammospheres were treated with B591 in the first generation and passaged two additional generations without B591 treatment. As shown in Fig. 4a, both the size and the number of primary mammospheres were reduced by B591 in a concentration-dependent manner. Furthermore, the cells derived from B591-treated primary mammospheres showed a significantly decreased sphere-forming ability in subsequent passages (Fig. 4b), indicating a reduced self-renewal capacity of CSCs after B591 treatment.

The effect of B591 on the CD44^high^/CD24^low^ population was examined in mammospheres from SUM-159PT cells. As shown in Fig. 4c, e, the presence of B591 significantly reduced the CD44^high^/CD24^low^ population in a dose-dependent manner and the treatment with 4 µM of B591 decreased the CD44^high^/CD24^low^ population by 82%. The effect of B591 on the breast CSCs population with high ALDH activity was further tested by flow cytometry. The results showed that B591 also significantly decreased the ALDH^high^ fractions and the treatment with 4 µM of B591 reduced the ALDH^high^ fractions by 90% in SUM-159PT cells (Fig. 4d, e).

A panel of breast CSCs related markers, including CSCs identification markers (ALDH1 and CD44), self-renewal markers (BMI-1, SOX-2, Oct-4, and Nanog) and epithelial-mesenchymal transition (EMT) markers (Vimentin, N-cadherin and Twist1), were examined by western blot analysis using lysate from DMSO or B591-treated SUM-159PT mammospheres (Fig. 4f). The results showed that B591 decreased the expression of these markers in a dose-dependent manner, indicating reduced population and inhibited self-renewal ability of CSCs after B591 treatment. Meanwhile, decreased phosphorylation status of downstream substrates of PI3K pathway, including S6K1, S6, and 4E-BP1, were detected in CSCs under B591 treatment (Fig. 4g).

The tumor-seeding ability is the putative functional characteristic of CSCs and change of this ability after B591 treatment was assessed according to the method previously described [36]. After treated with 4 μM of B591 for 24 h and subsequently allowed to recover in culture without treatment for 48 h, SUM-159PT cells were injected in serial limiting dilutions into the mammary fat pad of NOD/SCID mice, and B591 pretreatment resulted in a sevenfold decrease in tumor-initiating frequency (Fig. 4h). Collectively, these results indicated that B591 reduced the CSCs population in breast cancer cells in vitro.

### B591 suppresses tumor growth in vivo by eliminating breast CSCs

**Fig. 5:**
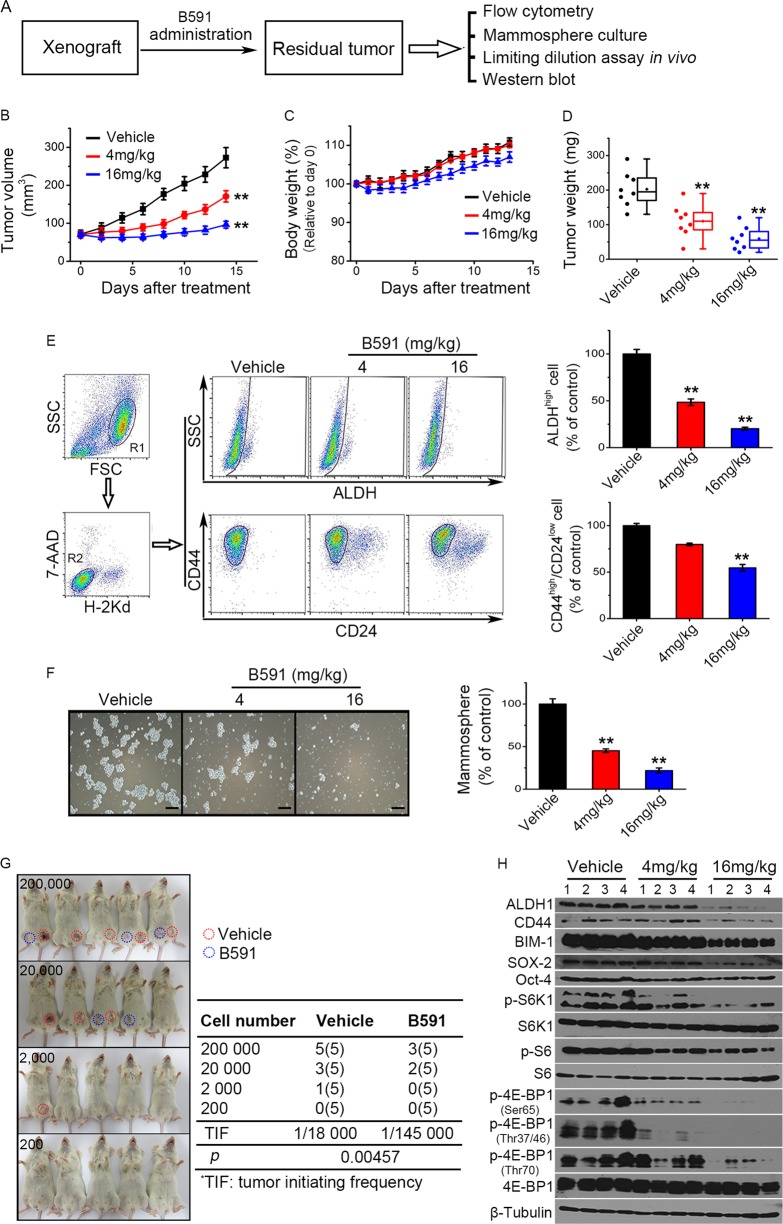
B591 abolishes tumor growth in vivo by eliminating CSCs. **a** MDA-MB-231 xenografts were treated with vehicle or B591 for 2 weeks, then the residual tumor tissue was harvested and dissociated into single cells for subsequent CSCs assays according with the flow chart. **b**–**d** Tumor weight and mice body weight were measured every 2 days during treatment. In the end of treatment, tumor tissues were collected and weighted (*n* = 8). **e** The CSCs markers of tumor cells were analyzed by flow cytometry. Only the live and non-mouse cell population was gated and analyzed. Data from three mice were plotted. **f** Mammosphere formation assay was carried out with the single cells. The relative mammosphere-formation ability is shown as percentage, and data represent mean ± SD. **g** The single cells from vehicle and 16 mg/kg of B591 group were replanted into NOD/SCID mice according to the indicating number, and 3 months later, tumors were counted for calculating the tumor-initiating frequency. **h** The CSCs markers and the downstream substrates of PI3K pathway were analyzed using the lysates from the primary xenografts by western blot analysis. ^**^p < 0.01, difference versus vehicle-treated control group

Then we evaluated the inhibitory effect of B591 on CSCs by in vivo xenograft assay (Fig. 5a). B591 (4 or 16 mg/kg) was given to the NOD/SCID mice bearing orthotopic xenografts of human breast cancer MDA-MB-231 cells for 2 weeks. Compared with vehicle treatment group, a remarkable decrease of tumor burden (determined by volume, 40% and 60% for 4 and 16 mg/kg dose group respectively) was observed under the treatment of B591 (Fig. 5b), with no significant body weight loss observed (Fig. 5c). Based on final tumor weights, 16 mg/kg B591 showed efficacy with 70.7% inhibition (Fig. 5d). The inhibition of PI3K/mTOR pathway by B591 in tumors was further confirmed by western blot analysis. As shown in Fig. 5h, B591 effectively decreased the phosphorylation levels of downstream substrates of PI3K pathway, including S6K1, S6, and 4E-BP1.

In the end of B591 treatment, the mice were killed, and tumors were harvested and dissociated into single cells for CSCs assay. The results showed that both the CD44^high^/CD24^low^ and the ALDH^high^ breast CSCs populations were significantly reduced by B591 (Fig. 5e). The reduction of CSCs was also proved by the decreased sphere-formation ability (Fig. 5f). Moreover, the decreased level of breast CSCs related markers, including CSCs identification marker (ALDH1 and CD44) and self-renewal markers (BMI-1, SOX-2, and Oct-4), further confirmed that B591 eliminated CSCs in vivo (Fig. 5h).

The primary tumor cells dissociated from xenografts were injected into the mammary fat pad of NOD/SCID mice in limiting dilutions. Cells from B591-treated tumors showed a sevenfold reduction of tumor-initiating frequency (Fig. 5g), demonstrating that B591 eliminated CSCs in primary tumors, thereby abrogating the initiation of secondary tumors.

### B591 reduces metastasis of breast cancer

**Fig. 6:**
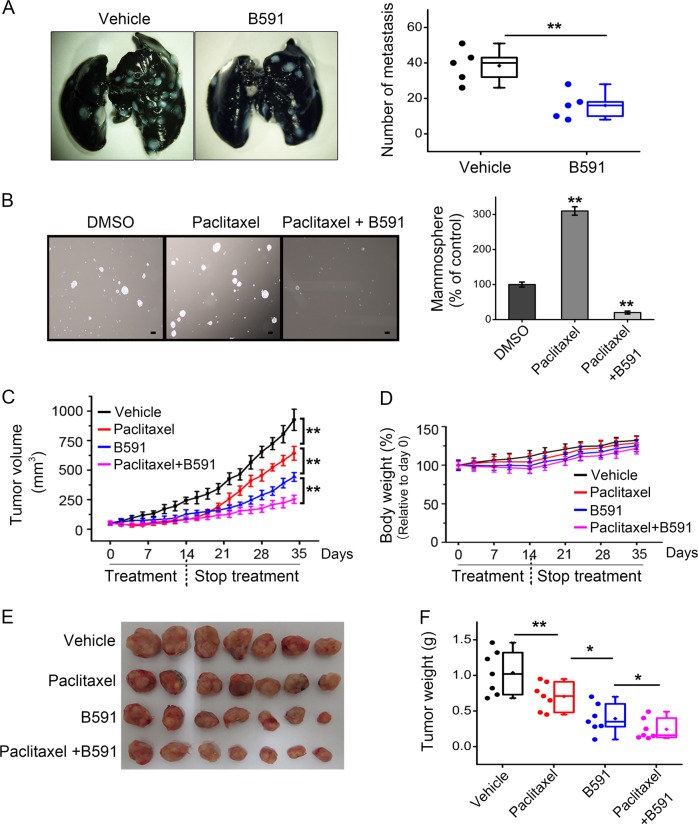
B591 reduces breast tumor metastasis and recurrence. **a** Representative images show the effect of B591 (8 mg/kg) on lung metastasis of 4T1 cells, and data from six mice were plotted. **b** SUM-159PT cells were respectively treated with the indicated agents (8 nM Paclitaxel, 8 nM Paclitaxel plus 5 μM B591) for 24 h, the cells were then cultured for mammospheres. Representative images were shown, and data from two independent experiments were plotted. **c**–**f** SUM-159PT xenografts (*n* = 7) were treated with 4 mg/kg Paclitaxel, or 10 mg/kg B591, or both for 2 weeks, then ended the treatment and waited for tumor recurrence. Tumor volume (**c**) and body weight (**d**) were measured every 2 days after treatment, and tumors were photographed (**e**) and weighted (**f**) at the end of the experiment. ^*^p < 0.05, ^**^p < 0.01 versus indicated groups

Studies implicated that the process termed EMT is associated with features of CSCs, which are considered to be involved in metastasis, a major cause of cancer-associated deaths [36–38]. 4T1 murine mammary carcinoma cells spontaneously produce highly metastatic tumors that can metastasize to the lung, liver, lymph nodes, and brain while the primary tumor is growing in situ [39, 40]. To determine the efficacy of B591 on tumor metastasis, the metastasis model was established by incubating 4T1 cells into the mammary gland of NOD/SCID mice and treated with 8 mg/kg of B591 intraperitoneally once a day. B591 decreased more than 50% of the metastatic tumors in the lung which spontaneously metastasize from the primary tumor in the mammary gland (Fig. 6a).

### B591 delays recurrence of chemo-treated breast cancer

CSCs exhibit increased resistance to chemotherapeutic agents and therefore have been postulated to account for tumor relapse after chemotherapy [34]. Based on the finding that B591 preferentially targets CSCs, we supposed that by eliminating the breast CSCs, B591 might delay breast tumor recurrence after initial treatment with chemotherapy drugs. Paclitaxel is a first-line chemotherapeutic agent used for breast cancer therapy. SUM-159PT cells were pre-treated with paclitaxel, and then the cells remaining alive were cultured for mammospheres. Consistent with the data of Fig. 3 and Supplementary Fig. S10, paclitaxel treatment enriched CSCs and hence increased the formation of mammospheres, while in the presence of B591, the paclitaxel-enriched CSCs were effectively eliminated (Fig. 6b).

Subsequently, we determined if B591 could delay tumor regrowth after initial treatment with paclitaxel in breast cancer xenograft model. In SUM-159PT xenografts, treatment with paclitaxel (4 mg/kg) for 2 weeks induced initial tumor remission, but these tumors regrew quickly after termination of paclitaxel therapy (Fig. 6c). B591 (10 mg/kg) caused significant inhibition of tumor growth and notably delayed the relapse of breast cancer following cessation of paclitaxel treatment, with no significant body weight loss observed (Fig. 6c–f). These results suggested great potential of B591 to improve the clinical therapy of cancer, especially combined with conventional chemotherapeutic drug paclitaxel.

## Discussion

PI3K/mTOR signaling pathway is one of the most frequently dysregulated pathways in human cancer and plays a critical role in driving tumor initiation and progression. Currently mTOR inhibitor rapalogs (such as temsirolimus and everolimus) and PI3K inhibitors (Copanlisib, a pan-class I PI3K inhibitor and Idelalisib, a PI3Kδ Inhibitor) are the only commercially available anti-cancer drugs targeting PI3K/mTOR pathway, validating PI3K and mTOR as crucial therapeutic targets in cancer [41–43]. In spite of preclinical and clinical therapeutic effect in selected tumor types, single agent rapalog therapy showed very limited anticancer efficacy in most tumor types. Hyper-activation of Akt and MAPK/ERK pathway by rapalogs has been proposed to be involved in this resistance. Inhibition of mTORC1 by rapalogs blocks the S6K-IRS1-Akt and S6K-PI3K-Ras feedback loops, leading to hyper-activation of PI3K signaling and MAPK activation [27, 28]. Consistently, our data showed that 24 h treatment of rapamycin on RD cells induced hyper-phosphorylation of both Akt and ERK. In contrast, B591, with its PI3K kinase inhibitory activity, displayed robust inhibition on the phosphorylation of both Akt and ERK, which is consistent with the finding that PI3K inhibitor, LY294002 or wortmanin, reduced Akt and ERK activation [27]. When compared with PI-103, a PI3K/mTOR dual inhibitor, B591 effectively inhibited the phosphorylation of ERK, consistent with the reports that PI-103 did not inhibit or even stimulated ERK phosphorylation [44, 45]. These results indicate that B591 has superior activity in overcoming the feedback activation of Akt and MAPK/ERK pathway than rapamycin and PI-103, predicting that B591 may achieve better clinical outcome than rapalogs or PI3K/mTOR dual inhibitors.

One recent study unraveled a PI3K-dependent mechanism for mTORC2 and its substrate Akt activation (S473). This study reported that PtdIns(3,4,5)*P*_3_, generated by activated PI3K, directly binds Sin1-PH to release its suppression on the mTOR kinase domain, thereby triggering mTORC2 activation [21]. This novel finding highlights the critical role of PI3K in governing mTORC2, which may well explain the potent effect of B591, a PI3K inhibitor, on suppressing both mTORC1 and mTORC2 (Fig. 2b), and preventing the feedback activation of Akt (Fig. 2e, f).

Growing evidence has demonstrated the critical role of PI3K/mTOR pathway in the maintenance of CSCs, and that targeting PI3K/mTOR signaling may be beneficial in cancer treatment by eliminating CSCs [12, 13, 46]. Therefore, several PI3K or PI3K/mTOR dual inhibitors have been designed and reported to preferentially target CSCs. Chang et al. showed that activation of PI3K/mTOR in prostate cancer led to enhanced CSCs phenotype and radio-resistance. While combination with BEZ235, a PI3K/mTOR dual inhibitor, radiotherapy markedly reduced CSCs and thus increased radio-sensitivity [47]. Kolev et al. reported that VS-5584, a PI3K/mTOR dual inhibitor which has been proceeded into clinical trials, preferentially reduced CSCs in breast cancer cell lines and xenografts. Triple knockdown of PI3Kα, PI3Kβ, and mTOR by siRNA displayed the strongest preferential reduction of CSCs, whereas knockdown of PI3Kα, PI3Kβ, or mTOR individually did not exert a preferential effect on CSCs, suggesting that simultaneous inhibition of PI3Kα and PI3Kβ isoforms and mTOR is important to exert a strong preferential effect on CSCs [48]. Consistently, B591, as a novel pan-PI3K kinase with all four PI3K isoforms inhibition, strongly suppressed the activity of mTORC1 and mTORC2 through inhibition of PI3K, and remarkably showed a preferential elimination on breast CSCs. These findings support the functional role of PI3K/mTOR pathway in the self-renewal and stem maintenance of breast CSCs, further suggesting that the PI3K/mTOR signaling pathway could be a promising target for development of CSCs targeting drugs.

Multiple, distinct subpopulations of breast CSCs have been identified using a variety of markers and functional attributes including CD44^high^/CD24^low^ and ALDH^high^ phenotype [5], implying that an ideal anti-CSCs drug should optimally target all CSCs pools. B591 appears to be such a candidate, as demonstrated in this study that B591 targets CSCs based on multiple independent assays, including CD44^high^/CD24^low^ CSCs marker assay, ALDH activity assay as well as western blot analysis of CD44 and ALDH. Moreover, B591 preferentially reduced the fraction of cells with self-renewal potential as detected by mammosphere formation assay in vitro and by limiting dilution assay in vivo of tumor initiating ability with replantation of tumor cells into secondary NOD/SCID mice. Growing evidence supports a critical role of CSCs in tumor metastasis, drug resistance, and recurrence [49]. As a CSCs targeting agent, B591 significantly reduces lung metastasis of breast tumor. This capability may be contributed by the fact that B591 inhibits the EMT (Fig. 4f) which is a key step toward cancer metastasis. Our comprehensive evaluation strongly demonstrated that B591 is an effective breast CSCs targeting agent.

With the advent of the CSCs hypothesis, intensive studies have been focused on identifying therapeutic strategies that selectively target CSCs, such as targeting specific CSC markers or key signaling cascades, targeting the CSC niche, or inducing apoptosis or differentiation in CSCs [50, 51]. However, for most advanced solid cancers, targeting a putative rare CSC population may have little effect on patient outcomes [52]. Hence, combining traditional chemotherapies with new CSCs targeting agents promises to be an efficient therapeutic strategy. Superior resistance to classical anticancer chemotherapies is known as one of the features of CSCs, predicting that agents that selectively target CSCs should function synergistically with chemotherapeutic drugs to delay tumor relapse [51]. Relevant to this point, our study showed that paclitaxel, a chemotherapeutic agent commonly used to treat breast cancer, enriched CSCs in SUM-159PT and MDA-MB-231 cells and hence increased the formation of mammospheres. While in the presence of B591, paclitaxel-enriched CSCs were effectively eliminated. In SUM-159PT xenografts, the combination B591 with paclitaxel significantly inhibited tumor growth and delayed the relapse of breast cancer following cessation of paclitaxel treatment. These data provided strong evidence that as a novel CSCs targeting agent, B591 overcame drug resistance to paclitaxel mediated by CSCs, and combining B591 with paclitaxel enhanced the therapeutic response and delays tumor recurrence. Our study thus provide a preclinical rationale to further evaluate B591 in combination with paclitaxel or other chemotherapies for breast cancer treatment in advanced preclinical and clinical settings.

Taken together, our findings provide compelling rationale for the further development of B591 as a CSCs targeting agent. Moreover, the discovery of B591 provides a novel chemical template for discovery and development of promising PI3K inhibitors, especially those preferentially targeting of CSCs.

## Materials and methods

### Chemicals and reagents

B591 was synthesized and identified according to Supplementary Materials and Methods (Supplementary Fig. S1-S4). For in vitro study, 10 mM stock solution of B591 in DMSO was used. For in vivo study, B591 was dissolved in the solution composed of 1.5% Tween-20, 5% PEG400 and 2% Cremophor EL in 0.9% Saline. Rapamycin, PI-103 and paclitaxel were purchased from Selleckchem (Houston, TX, USA).

### In vitro PI3K kinase assay

Purified PI3K Class I Lipid Kinase, including p110α/p85α, p110β/p85α, p110γ, and p110δ/p85α (Promega, Madison, WI, USA), was respectively incubated with B591 for 30 min at room temperature. The activity of PI3K was evaluated using a PI3-Kinase Activity ELISA kit (K-1000s, Echelon, Salt Lake City, UT, USA) according to the manufacture’s instructions. The assay is a competitive ELISA in which the signal is inversely proportional to the amount of PI(3,4,5)P_3_ produced. After the PI3-Kinase reactions are complete, reaction products are first mixed and incubated with a PI(3,4,5)P_3_ detector protein, then added to the PI(3,4,5)P_3_-coated microplate for competitive binding. A peroxidase-linked secondary detector and colorimetric detection is used to detect PI(3,4,5)P_3_ detector binding to the plate. The colorimetric signal is inversely proportional to the amount of PI(3,4,5)P_3_ produced by PI3-Kinase. The absorbance was measured at 450 nm with a Multilabel Reader (PerkinElmer, Waltham, MA, USA). ATP competition assays were executed with varying concentrations of ATP, and five concentrations of ATP were 5 μM, 25 μM, 50 μM, 100 μM, and 200 μM respectively. The IC_50_ values for inhibition of PI3Kβ by B591 were determined at indicated concentrations of ATP. In addition, B591 selectivity was tested against 39 kinases at Life Technologies.

### Cellular PI3K kinase assay

Cells were seeded at density of 3 × 10^7^ cells/150 mm dish. After treatment with different concentrations of B591 for 10 h, the media was removed and 10 ml of ice-cold 0.5 M TCA was immediately added. Incubate cells on ice for 5 min. Cells were scraped and centrifuged at 3000 rpm for 7 min at 4 ℃. The pellet was resuspended in 3 ml of 5% tricarboxylic acid/1 mM EDTA, vortexed for 30 s, and centrifuged at 3000 rpm for 5 min. The supernatant was discarded and the cells was washed one more time. Afterward, neutral lipids were extracted adding 3 ml of MeOH:CHCl_3_ (2:1) and continuously vortexing for 10 min at room temperature. Extracts were centrifuged at 3000 rpm for 5 min, the supernatant was discarded, and this extraction step was repeated one more time. The acidic lipids were extracted adding 2.25 ml MeOH:CHCl_3_:12 M HCl (80:40:1) with continuous vortexing over 25 min at room temperature. Extracts were centrifuged at 3000 rpm for 5 min and the supernatant was transferred to a new 15 ml tube; 0.75 ml of CHCl_3_ and 1.35 ml of 0.1 M HCl were added to the supernatant, vortexed, and centrifuged at 3000 rpm for 5 min to separate organic and aqueous phases. The organic (lower) phase was collected; 1.5 ml were transferred into positive displacement pipette and dried in a vacuum dryer. PIP_3_ samples and PI(4,5)P_2_ samples were resuspended in PBS-Tween + 3% Protein Stabilizer (provided by the Echelon kit), and were sonicated in an ice-water bath for 10 min, vortexed, and spun down before adding to the ELISA. All experiments were performed at least three times, each carried out in biological triplicate. Once phospholipids were isolated from cells, PIP_3_ and PI(4,5)P_2_ levels were measured using ELISA kits (Echelon, K-2500s and K4500) according to the manufacturer’s instructions.

### Mammosphere formation assay

As reported earlier [53], cells were cultured in 96-well Ultra-Low attachment plates (250 cells/well) with serum-free medium supplemented with 1 × B27 Supplement (Thermo Fisher Scientific, Sunnyvale, CA, USA), 1 × 2-Mercaptoethanol, 5 μg/ml insulin (Thermo), 1 μg/ml hydrocortisone (Sigma-Aldrich, Saint-Louis, MO, USA), 20 ng/ml human epidermal growth factor (Thermo), 10 ng/ml fibroblast growth factor (Thermo) and 0.5% Methyl cellulose (Sigma-Aldrich). TrypLE Express (Thermo) was used for passaging mammospheres.

In mammosphere formation assay, mammospheres were cultured in the presence of B591 for 5 days as described above and then were counted and imaged under a phase-contrast microscopy (Nikon, Japan).

In mammosphere passage assay, mammospheres were cultured as described above and treated with B591 in the first generation and passaged two additional generations without B591 treatment. For passaging, mammospheres were collected by centrifuge (1000 g, 5 min, 4℃) and dissociated by TrypLE Express (Thermo), then the mixture was passed through a cell strainer (40-micron, Corning) to obtain the single cell suspension for subsequently mammosphere formation as described above.

### CSCs phenotype assay and ALDH activity assay

Cells were treated with B591 for 24 h, then the cell suspension was incubated with 20 μl of CD44-APC (#559942, BD Pharmingen, Franklin Lakes, NJ, USA) and 20 μl of CD24-PE (#555428, BD Pharmingen) antibodies for 30 min in dark at 4 ℃. Phenotype was detected by BD FACSCalibur, and data was analyzed with Flowjo. ALDH activity was analyzed with ALDEFLUOR Kit (STEMCELL Technologies, Vancouver, BC, Canada) according to the manufacture’s protocol.

### CSCs study in vivo

Four-week-old female NOD/SCID mice (Beijing Vital River Laboratory Animal Technology Co., Ltd.) were used as host in all the CSCs studies in vivo. All animal procedures were conducted under the guidelines approved by the Animal Ethics Committee of Kunming Institute of Botany (Kunming, China). In limiting dilution assay, SUM-159PT cells were pretreated with 4 μM B591 for 24 h and then cultured for 48 h without B591. The live cells were collected and counted, the same amount of control cells and B591-treated cells were then incubated into the fourth left and right mammary fat pad of mice, respectively, according the indicated number that is 5 × 10^3^, 5 × 10^4^, and 5 × 10^5^ for each group. Tumors were inspected 3 months later, and the tumor initiating frequency (TIF) was calculated with ELDA software [54].

In MDA-MB-231 xenografts experiment, 1 × 10^6^ cells were incubated into the mammary fat pad of mice orthotopically. When the volume of tumor reached above 50 mm^3^, the mice were randomly divided into three groups for introperitoneal (i.p.) injection with vehicle, 4 mg/kg and 16 mg/kg B591. The tumor volume and the body weight of mice were measured during the 2-weeks treatment. In the end of treatment, the mice were killed, and the tumor tissues were dissected and dissociated into single cell suspension with Dispase II (Roche, Basel, Switzerland) according with the manufacture’s protocol. Then these single cells were used for CSCs phenotype assay, ALDH activity assay, mammosphere formation assay and limiting dilution assay in vivo as described above.

In the tumor recurrence study, orthotopic xenografts were established with 5 × 10^6^ SUM-159PT cells. When tumor volume reached about 50 mm^3^, mice were randomly divided into four groups for treatment with vehicle, 4 mg/kg Paclitaxel, 10 mg/kg B591, and combination of 4 mg/kg Paclitaxel plus 10 mg/kg B591 (i.p.). Treatment was stopped after 2 weeks, and the tumors were allowed for recurrence for 3 weeks. Tumor volume and the body weight of mice were measured during the experiments. In the end of treatment, the mice were killed, and the tumor tissues were dissected and weighted.

### Breast cancer metastasis study

To establish the metastasis model, 3 × 10^5^ of 4T1 cells were incubated into the mammary fat pad of NOD/SCID mice. When tumor volume reached about 50 mm^3^, mice were randomly divided into two groups for treatment with 8 mg/kg of B591 or vehicle (i.p.) for 2 months. The metastatic tumors in lung were stained with ink as previously reported [55].

### Immunofluorescence and high content assay

A detailed description is given in Supplementary Information.

### Western blot analysis

A detailed description is given in Supplementary Information.

### Statistical analysis

For in vivo studies, the data were expressed as mean values ± standard error (mean ± SE). The other results were expressed as mean ± standard deviation (mean ± SD). All statistical analyses were performed using an unpaired *t*-test. A *p* value of less than 0.05 was considered to be significant.

## Supplementary information

Supplementary Information
