# Supplementary material for: B591, a novel specific pan-PI3K inhibitor, preferentially targets cancer stem cells
Source: Oncogene. 2019 Jan 11;38(18):3371–86. doi: 10.1038/s41388-018-0674-5 (PMC6756013; doi:10.1038/s41388-018-0674-5)
Supplement: Supplementary file 1 — Supplementary Information [file 41388_2018_674_MOESM1_ESM.doc]

**Supplementary Information**

**Table of contents**

**I. Supplementary methods**

1. General Experimental

2. Synthesis Procedures and Analytical Data

3. Crystal data for **B591**

4. Cell lines and cell culture

5. Chemicals and Reagents

6. Physicochemical and ADME properties estimation

7. Western blot analysis

8. Immunofluorescence and high content assay

9. Cell viability assay

10. Cell cycle analysis

11. Apoptosis assay

12. Tumor initiation assay *in vivo*

13. qPCR assay

**II. Supplementary results**

1. Supplementary Fig. S1 Synthesis of dihydrobenzofuran–imidazolium salts.

2. Supplementary Fig. S2 ^1^H NMR spectrum of B591.

3. Supplementary Fig. S3 ^13^C NMR spectrum of B591.

4. Supplementary Fig. S4 X-ray crystallographic structure of B591.

5. Supplementary Fig. S5. B591 inhibits PI3K/mTOR signaling pathway.

6. Supplementary Fig. S6. B591 prevents feedback activation of Akt and ERK in MDA-MB-231 cells.

7. Supplementary Fig. S7. B591 inhibits tumor cell proliferation.

8. Supplementary Fig. S8. B591 induces G0/G1 cell cycle arrest and apoptosis in tumor cells.

9. Supplementary Fig. S9. CSCs are enriched in mammospheres.

10. Supplementary Fig. S10. B591 preferentially targets CSCs *in vitro*.

11. Supplementary Table S1. Biochemical kinase profiling data of B591 against 39 human kinases (Life Technologies).

12. Supplementary Table S2a. Physicochemical parameters of B591 estimated by Molinspiration server.

13. Supplementary Table S2b. ADME, bioavailability and synthetic accessibility properties of B591.

14. Supplementary Table S3. Sequences of qPCR primers.

**I. Supplementary methods**

**1. General Experimental**

Melting points were obtained on a XT-4 melting-point apparatus and were uncorrected. Proton nuclear magnetic resonance (^1^H-NMR) spectra were recorded on a Bruker Avance 300 spectrometer at 300 MHz. Carbon-13 nuclear magnetic resonance (^13^C-NMR) was recorded on Bruker Avance 300 spectrometer at 75 MHz. Chemical shifts are reported as δ values in parts per million (ppm) relative to tetramethylsilane (TMS) for all recorded NMR spectra. Low-resolution Mass spectra were recorded on a VG Auto Spec-3000 magnetic sector MS spectrometer. High Resolution Mass spectra were taken on AB QSTAR Pulsar mass spectrometer.

Silica gel (200–300 mesh) for column chromatography and silica GF_254_ for TLC were produced by Qingdao Marine Chemical Company (China). All air- or moisture- sensitive reactions were conducted under an argon atmosphere. Starting materials and reagents used in reactions were obtained commercially from Acros, Aldrich, Fluka and were used without purification, unless otherwise indicated.

**2. Synthesis Procedures and Analytical Data**

**2.1 Synthesis of compound 2**

POCl_3_ (85mmol) was added in small portions with slight warming into a solution of dihydrobenzofuran **1** (43 mmol) in anhydrous DMF (92 mmol). The mixture was heated for 6 h at 80 °C. The cooled mixture was treated with NaOH solution in water (1.0 M) until the pH rose to 7. The organic compounds were extracted with EtOAc (3 × 20 ml). The combined organic layers were washed with saturated KHCO_3_ solution (3 × 10 ml) and water (20 ml), then dried over anhydrous Na_2_SO_4_ and concentrated. The residue was purified by column chromatography (silica gel, petroleum ether 60–90 ^o^C : ethyl acetate = 5 : 1) to afford the product **2** (93%) as yellow oil.

Yield 93%. Yellow oil. ^1^H NMR (300 MHz, CDCl_3_) δ: 9.83 (s, 1H), 7.75 (d, 1H, *J* = 1.0 Hz), 7.67 (dd, 1H, *J* = 1.0, 8.4 Hz), 6.88 (d, 1H, *J* = 8.4 Hz), 4.70 (t, 2H, *J* = 8.8 Hz), 3.28 (t, 2H, *J* = 8.8 Hz). ^13^C NMR (75 MHz, CDCl_3_) δ: 190.5, 165.6, 132.9, 130.4, 128.5, 125.9, 109.5, 72.4, 28.7.

**2.2 Synthesis of compound 3**

To a stirred solution of dihydrobenzofuran-5-carbaldehyde **2** (28.2 mmol) in MeOH (25 mL) at 0 °C was added NaBH_4_ (56.4 mmol, 2.0 eq) in small portions over a period of 20 minutes, and then at ambient temperature for 4 h. Reaction progress was monitored by TLC. A small amount of water was added and the mixture was stirred for 15 min before rotary evaporation. The solvent was evaporated under reduced pressure and the residue was chromatographed on silica gel (petroleum ether 60-90 °C : ethyl acetate = 3:1) to afford the products **3** (90%) as white oil.

Yield 90%. White oil.  ^1^H NMR (300 MHz, CDCl_3_) δ: 7.71 (d, 1H, *J* = 1.0 Hz), 7.62 (dd, 1H, *J* = 1.0, 8.4 Hz), 6.83 (d, 1H, *J* = 8.4 Hz), 4.71 (t, 2H, *J* = 8.8 Hz), 4.65 (s, 2H), 3.24 (t, 2H, *J* = 8.8 Hz). ^13^C NMR (75 MHz, CDCl_3_) δ: 165.5, 132.4, 130.1, 128.1, 125.4, 109.6, 72.1, 69.8, 28.7.

**2.3 Synthesis of compound 4**

To a solution of dihydrobenzofuran 5-methanol compound **3** (32 mmol) in dichloromethane (50 mL) was added methanesulfonyl chloride (48 mmol, 1.5 eq) and triethylamine (96 mmol, 3.0 eq) at 0 ^o^C. The resulting mixture was stirred at room temperature for 2 h. After quenching the reaction with water (50 mL), the layers were separated. The organic phase was dried over anhydrous Na_2_SO_4_ and concentrated, and used for the next synthetic step. A mixture of the previous methanesulfonate and various substituted imidazole (imidazole, 2-methyl-imidazole, benzoimidazole, 2-methyl-benzoimidazole or 5,6-dimethyl-benzoimidazole) (96 mmol, 3.0 eq) was stirred in 1,4-dioxane (20 ml) at 105 ^o^C for 24 h (monitored by TLC). After cooling to room temperature, the solvent was concentrated, and the residue was diluted with EtOAc (20 mL). The organic layer was washed with water (20 mL) and brine (20 mL), dried over anhydrous Na_2_SO_4_ and concentrated. The residue was purified by column chromatography (silica gel, petroleum ether 60–90 ^o^C : ethyl acetate : Et_3_N = 1 : 1 : 0.2) to afford **4a-4e** in 60-75% yield (two steps).

1-((2,3-Dihydrobenzofuran-5-yl)methyl)-5,6-dimethyl-1*H*-benzo[*d*]imidazole **(4e)**. Yield 72%. White powder, mp 103-105 ^o^C. IR νmax (cm^-1^): 3430, 3042, 2952, 1612, 1493, 1352, 1232, 1146, 1094, 982, 935, 813, 741, 667. ^1^H NMR (300 MHz, CDCl_3_): δ 7.78 (s, 1H), 7.56 (s, 1H), 7.07 (s, 1H), 6.98 (d, 1H, *J* = 7.8 Hz), 6.96 (s, 1H), 6.73 (d, 1H, *J* = 7.8 Hz), 5.18 (s, 1H), 4.53 (t, 2H, *J* = 8.8 Hz), 3.11 (t, 2H, *J* = 8.8 Hz), 2.35 (s, 3H), 2.34 (s, 3H). ^13^C NMR (75 MHz, CDCl_3_): δ 160.1, 142.7, 142.34, 132.5, 132.1, 131.0, 128.0, 127.7, 127.3, 123.9, 110.1, 109.4, 71.4, 48.5, 29.6, 20.6, 20.2. HRMS (ESI-TOF) *m*/*z* Calcd for C_18_H_18_N_2_ONa [M+Na]^+^ 301.1317, found 301.1315.

**2.4 Synthesis of dihydrobenzofuran–imidazolium salt (B591)**

A mixture of dihydrobenzofuran–imidazole hybrid **4** (1 mmol) and phenacyl and alkyl halides (1.2 mmol, 1.5 eq) was stirred in 1,4-dioxane (10 ml) at 105 ^o^C for 24 h. An insoluble substance was formed. After completion of the reaction as indicated by TLC, the precipitate was filtered through a small pad of Celite, and washed with 1,4-dioxane (3 × 10 ml), then dried to afford dihydrobenzofuran–imidazolium salts **5-47** in 69-93% yields.

3-(2-bromobenzyl)-1-((2,3-dihydrobenzofuran-5-yl)methyl)-5,6-dimethyl-1H-benzo[d]imidazol-3-ium bromide **(B591)**. Yield 84%. White powder. IR *ν*_max_ (cm^-1^): 3412, 3096, 2957, 2745, 1834, 1612, 1552, 1489, 1439, 1351, 1237, 1120, 982, 939, 853, 760, 667, 617. ^1^H NMR (300 MHz, CDCl_3_): δ 11.30 (s, 1H), 7.59 (d, 1H, *J* = 7.5 Hz), 7.56 (d, 1H, *J* = 6.6 Hz), 7.46 (d, 1H, *J* = 5.4 Hz), 7.21-7.36 (m, 4H), 6.72 (d, 1H, *J* = 8.1 Hz), 5.89 (s, 2H), 5.76 (s, 2H), 4.53 (t, 2H, *J* = 8.8 Hz), 3.16 (t, 2H, *J* = 8.8 Hz), 2.36 (s, 3H), 2.33 (s, 3H). ^13^C NMR (75 MHz, CDCl_3_): δ 160.8, 142.00, 137.4, 133.4, 131.8, 131.0, 129.9, 129.7, 128.7, 128.6, 125.5, 124.7, 123.6, 113.5, 113.3, 109.5, 71.6, 51.2, 51.1, 29.5, 20.6. HRMS (ESI-TOF) *m/z* Calcd for C_25_H_24_BrN_2_O [M-Br]^+^ 447.1067, found 447.1066.

**3. Crystal data for B591.**

C_28_H_27_Br_2_N_2_O, *M* = 603.75, Monoclinic, *a* = 23.785(3) Å, *b* = 13.1745(16) Å, *c* = 15.3308(19) Å, *α* = 90.00°, *β* = 107.432(2)°, *γ* = 90.00°, *V* = 4583.4(10) Å^3^, *T* = 293(2) K, space group *P2(1)/c*, *Z* = 7, *μ*(MoKα) = 0.71073 mm^-1^, 25720 reflections measured (3.58° ≤ 2Θ ≤ 50°), 8069 unique (R_int_ = 0.0799, R_sigma_ = N/A) which were used in all calculations. The final *R_1_* values were 0.0508 (*I* > 2*σ*(*I*)). The final *wR*(*F*^2^) values were 0.0999 (*I* > 2*σ*(*I*)). The final *R_1_* values were 0.1299 (all data). The final *wR*(*F*^2^) values were 0.1181 (all data). The goodness of fit on *F*^2^ was 0.886.

**4. Cell lines and cell culture**

SW480 cell line was purchased from American Type Culture Collection and SUM-159PT cell line was obtained from Asterand. A549, MCF7, HCT116, SMMC7721, HepG2, MDA-MB-468, RD, and 4T1 cells were purchased from Cell Bank of Type Culture Collection of Chinese Academy of Sciences (Shanghai, China); Recombinant CHO-hIR cells stably expressing human Akt1 fused to the C-terminus of enhanced green fluorescent protein (EGFP) was purchased from Thermo scientific (Logan, Utah, USA); Cells authenticated by suppliers were expanded and stored in liquid nitrogen once receiving each cell line. MDA-MB-231 cell line was kindly gifted from Dr. Shile Huang (Louisiana State University Health Sciences Center, Shreveport, United States). All cell lines were cultured according to the suppliers’ instructions and passaged no more than two months.

**5. Chemicals and Reagents**

RPMI 1640, Dulbecco’s Modified Eagle Medium (DMEM) and fetal bovine serum

(FBS) were purchased from HyClone (Logan, UT, USA). Recombinant human IGF-1 was purchased from Peprotech. Antibodies of phospho-Akt at T308 (#9275), phospho-Akt at S473 (#4060), phospho-S6K1 at T389 (#9206), S6K1 (#2708), phospho-S6 at S235/236 (#2211), 4E-BP1 (#9452), phospho-4E-BP1 at S65 (#9451), phospho-4E-BP1 at T37/46 (#9459), phospho-4E-BP1 at T70 (#9455), phospho-GSK3β at S9 (#9336), GSK3β (#9832), ALDH1 (#12035), BMI-1 (#6964), Oct-4 (#2890), CD44 (#5640), SOX-2 (#2748)，Nanog (#4903), p27 (#2552) and cleaved caspase-3 (#9664) were purchased from Cell Signaling Technology (Boston, MA, USA). Antibodies of Akt1 (sc-5298), S6 (sc-74459), p-ERK (sc-7383), Vimentin (sc-32322), Twist1 (sc-15393), cyclinD1 (sc-20044), PARP (sc-8007), Bcl-XL (sc-8392), caspase-3 (sc-56052), Cleaved-caspase-3 (#9664), β-actin (sc-81178) and β-Tubulin (sc-5274) were from Santa Cruz Biotechnologies (CA, USA). Antibodies of N-cadherin (610920) and eIF4E (610269) were obtained from BD Biosciences (San Jose, CA, USA). The secondary antibodies of anti-Mouse IgG-Peroxidase (A9044) and anti-Rabbit IgG-Peroxidase (A0545) were from Sigma-Aldrich (Saint-Louis, MO, USA).

**6. Physicochemical and ADME properties estimation**

The estimation of the physicochemical property of B591 was performed using Molinspiration online server (http://www.molinspiration.com/). The pharmacokinetics, bioavailability and synthetic accessibility properties of B591 were estimated by using robust SwissADME server.

**7. Western blot analysis**

The cells were treated with DMSO or indicated compounds. Western blot analysis was performed as described previously [1]. Specific proteins were incubated with enhanced chemiluminescence reagent and detected using Luminescent Image Analyzer LAS-4000 mini System (GE Healthcare, USA).

In *in vivo* studies, tumor samples were collected, flash frozen in liquid nitrogen, and stored at -80℃ until processed. The cell lysates were prepared in lysis buffer (50 mM Tris pH 7.6, 0.2 mM EDTA, 10 mM MgCl_2_, 0.2% Triton X-100, protease inhibitor cocktail) and homogenized on ice. The protein extracts were boiled for 5 minutes and were stored at -20℃. The protein concentration was determined by BCA assay (Thermo Scientific), and 20 μg total proteins were loaded for immunoblotting. Western blot analysis was performed as described previously [1].

**8. Immunofluorescence and high content assay**

Cells were seeded in 96-well plates (5,000 cells/well) and treated with DMSO or B591 for 5 h. Cells were then fixed with 4 % paraformaldehyde and followed by permeabilization with 0.1% triton X-100 and blocking in 2% bovine serum albumin in PBS. Cells were prepared for immunofluorescence by incubation with the anti-eIF4E antibody overnight and washed in PBS followed by incubation with anti-mouse IgG Alexa Fluor® 546 antibody (Invitrogen, Carlsbad, CA, USA). The subcellular location of eIF4E and the eIF4E nuclear–cytoplasmic intensity of each field per well was monitored and analyzed by using ArrayScan® VTI HCS Reader according to manufacturer’s instruction.

**9. Cell viability assay**

Cell viability in various cancer cell lines was determined by MTS (3-(4,5-dimethylthiazol-2-yl)-5-(3-carboxymethoxyphenyl)- 2-(4-sulfophenyl)-2H-

tetrazolium, inner salt) assay in 96-well plates according to the protocol of CellTiter 96® AQueous One Solution Cell Proliferation Assay kit (Promega, Madison, USA). Briefly, 100 µl of cell suspensions were seeded into each well of 96-well plate and allowed to adhere for 24 hours. Cancer cells were exposed to different concentrations of B591 in triplicates for 48 hours. 20 μl of CellTiter 96® AQ_ueous_ One Solution Reagent was added in each well and the cells were further incubated at 37 ºC for 1-2 hours. Cell viability was detected by measuring the absorbance at a wavelength of 490 nm. Concentrations of 50% inhibition of cell viability (IC_50_) were determined on the basis of the relative survival curve.

**10. Cell cycle analysis**

MDA-MB-231 cells were seeded in six-well plates at a density of 4×10^5^ cells per well. The next day, cells were treated with B591 (0, 2.5, 5, 10 μM) for 24 h. Then the cells were trypsinized, centrifuged at 300 g for 5 minutes and fixed in 1 ml of 70 % cold ethanol. After incubation at -20℃ overnight, the cells were collected by centrifugation at 500 g for 5 min and pellets were resuspended in 500 μl PBS containing 300 μg/ml of RNase (Sigma, MO, USA). After incubation at 37 ℃ for 30 min, the pellets were stained with 100 μg/ml of propidium iodine (Sigma, MO, USA) for 20 min. Cell cycle distribution was determined by flow cytometry (FACScaliber, Becton Dickinson).

**11. Apoptosis assay**

Cells were seeded in six-well plates at a density of 2×10^5^ cells per well and kept overnight at 37℃ humidified incubator with 5% CO_2_. The next day, cells were treated with B591 for 48 h. Cells were then collected and stained using Annexin V-FITC Apoptosis Detection Kit I (BD Pharmingen™, Franklin Lakes, NJ, USA) according to manufacturer’s instruction. In brief, cells were washed with cold PBS twice and with binding buffer once, about 1×10^5^ cells were resuspended in 100 μl Annexin-V binding buffer, followed by incubation with FITC conjugated Annexin V and propidium iodide (PI) for 15 min at room temperature in the dark. The apoptosis assay was performed by using BD FACSCalibur flow cytometry.

**12. Tumor initiation assay *in vivo***

1000 cells derived from SUM-159PT mammosphere and monolayer culture were incubated into the fourth left and right mammary fat pad of 4 NOD/SCID mice, respectively. Tumors were inspected 3 months later, and the tumor initiating frequency was recorded.

**13. qPCR assay**

qPCR assay was carried according with previously reported [2]. Total RNA was prepared with TRIzol (ThermoFisher) according to the manufacturer´s protocol. Reverse transcription was performed using RevertAid H Minus First Strand cDNA Synthesis Kit (ThermoFisher). For qPCR, SYBR Select Master Mix (ThermoFisher) was used with ABI 7500 Real-Time PCR System. mRNA from SUM-159PT mammosphere and monolayer culture were analyzed. All primers were listed on supplementary table S3.

**II. Supplementary results**

**Supplementary Fig. S1**

**Supplementary Fig. S1.** Synthesis of dihydrobenzofuran–imidazolium salts.

**Supplementary Fig. S2**

**Supplementary Fig. S2.** ^1^H NMR spectrum of **B591**.

**Supplementary Fig. S3**

**Supplementary Fig. S3.** ^13^C NMR spectrum of **B591**.

**Supplementary Fig. S4**

**Supplementary Fig. S4.** X-ray crystallographic structure of **B591**.

**Supplementary Fig. S5**

**
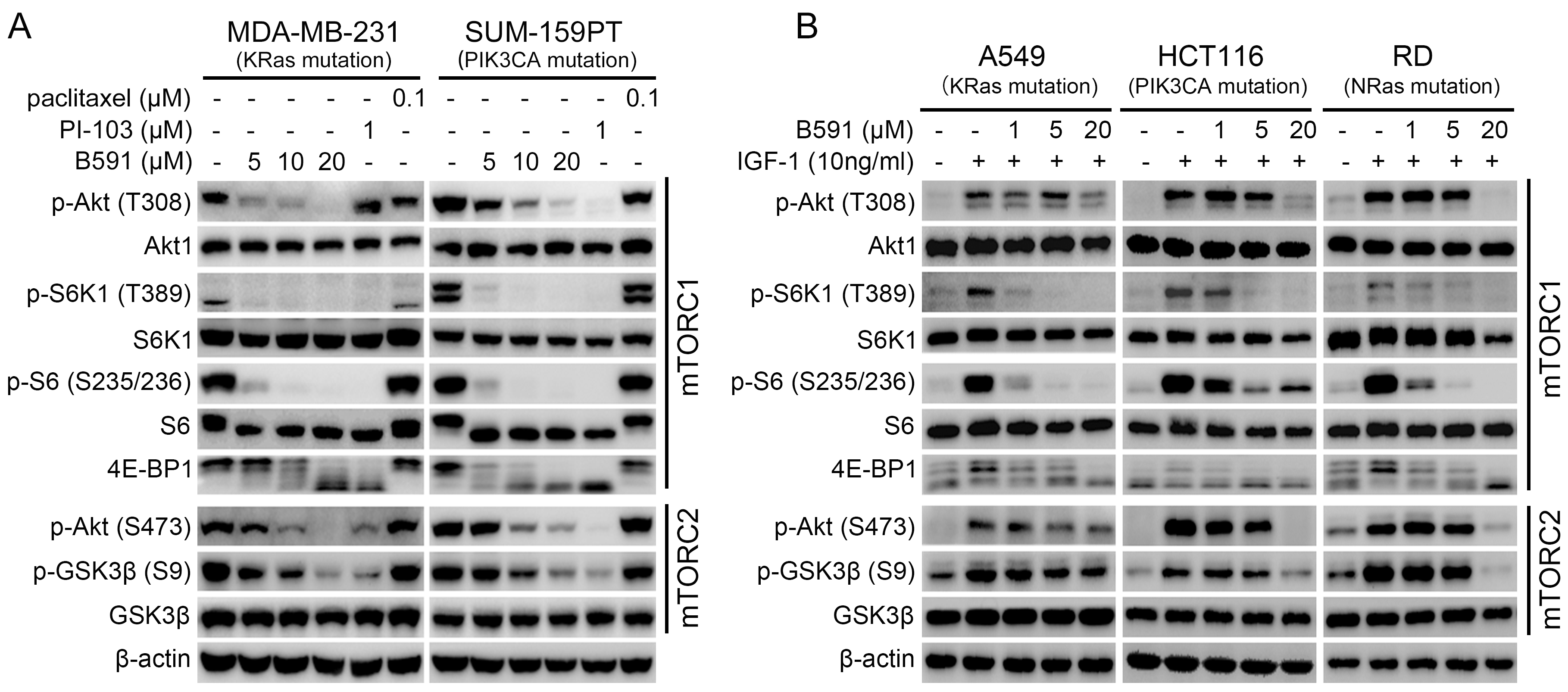
**

**Supplementary Fig. S5. B591 inhibits PI3K/mTOR signaling pathway.** (A) MDA-MB-231 and SUM-159PT cells were treated with indicated concentrations of B591, PI-103 or paclitaxel for 5 h, the cells were harvested and cellular extracts were subjected to Western blot analysis. (B) Serum-starved A549, HCT116 and RD cells were treated with indicated concentrations of B591 and then stimulated with 10 ng/ml of IGF-I for 1 h, followed by Western blot analysis with indicated antibodies. β-actin was used as a loading control.

**Supplementary Fig. S6**

**
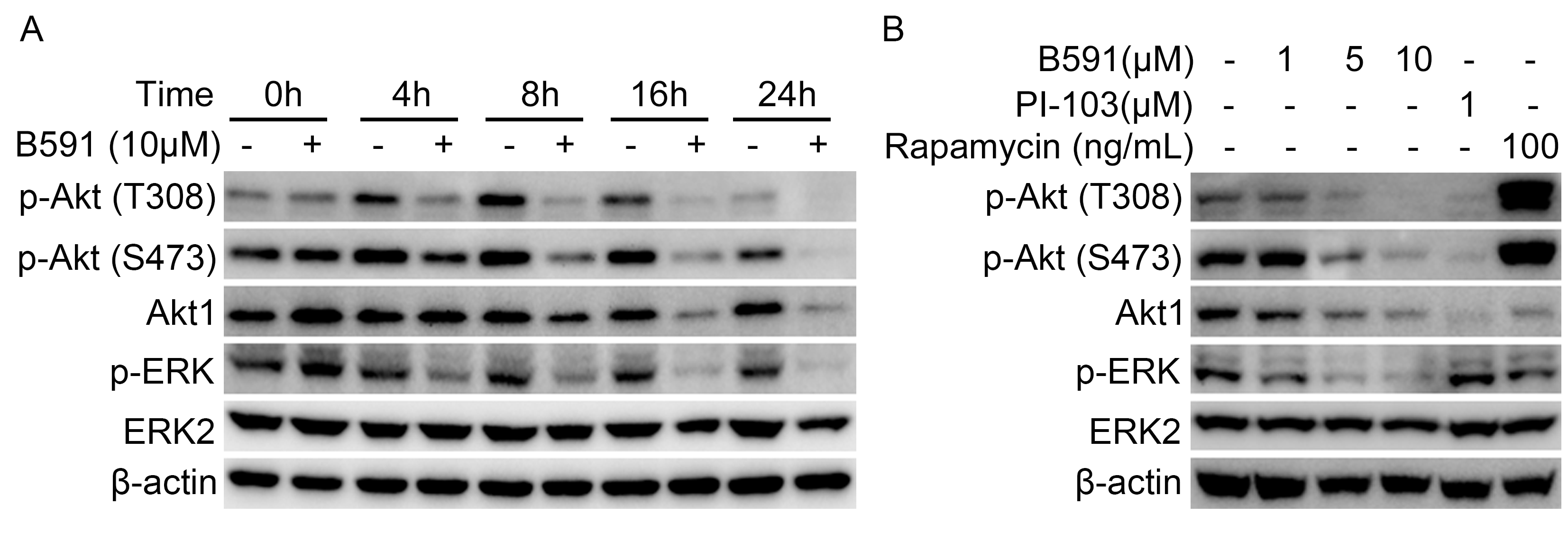
**

**Supplementary Fig. S6. B591 prevents feedback activation of Akt and ERK in MDA-MB-231 cells.** (A) The phosphorylation of AKT and ERK was determined by immunoblotting in MDA-MB-231 cells in the presence of 10 μM of B591 after indicated time of exposure. (B) Inhibition of the phosphorylation of AKT and ERK in MDA-MB-231 cells treated with B591, PI-103 or Rapamycin for 24 h was determined by immunoblotting. β-actin was used as a loading control.

**Supplementary Fig. S7**

**
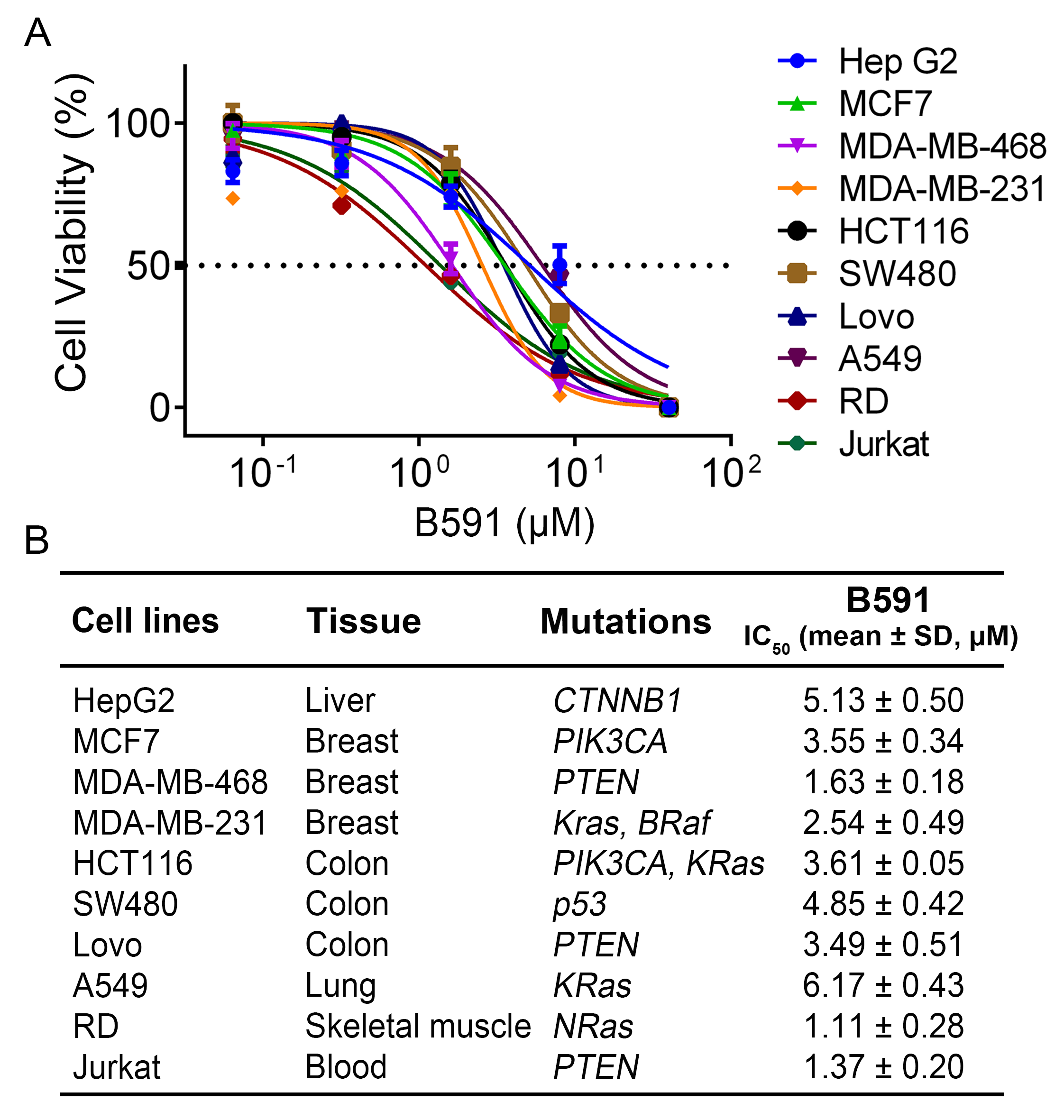
**

**Supplementary Fig. S7. B591 inhibits tumor cell proliferation.** (A) The effect of B591 on cell viability in a panel of cells derived from different cancer types with different mutation status. Cells were treated with increasing concentrations of B591 for 48 h and cell viability was detected by MTS assay. (B) The anti-proliferative potency of B591 against different cancer cells. IC_50_ values were shown as mean ± SD.

**Supplementary Fig. S8**


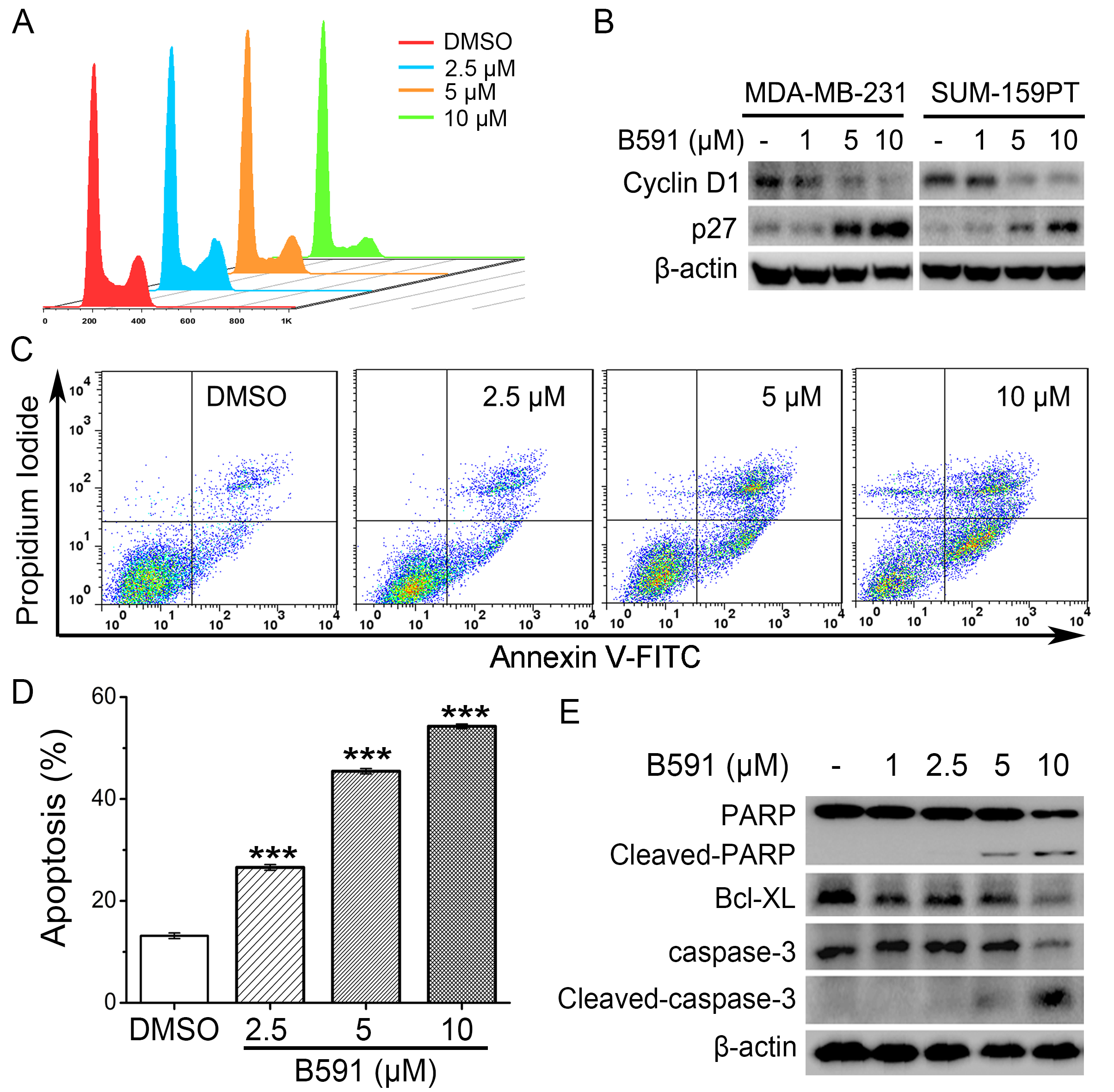


**Supplementary Fig. S8. B591 induces G0/G1 cell cycle arrest and apoptosis in tumor cells.** (A) MDA-MB-231 cells were treated with indicated concentrations of B591 for 24 h. The cells were harvested and analyzed for cell cycle profile. (B) MDA-MB-231 and SUM-159PT cells were treated with indicated concentrations of B591 for 24 h, the cells were harvested and cellular extracts were subjected to Western blot analysis. β-actin was used as a loading control. (C) Human rhabdomyosarcoma RD cells were treated with indicated concentrations of B591 for 48 h. The cells were harvested and processed for apoptosis assay using the Annexin V-FITC Apoptosis Detection Kit. (D) Quantification of flow cytometry analysis of apoptosis. Results were presented as mean ± SD (n = 3). ^***^p <0.001, difference versus DMSO-treated control group. (E) RD cells were treated with indicated concentrations of B591 for 24 h, the cells were harvested and cellular extracts were subjected to western blot analysis. β-actin was used as a loading control.

**Supplementary Fig. S9**

**
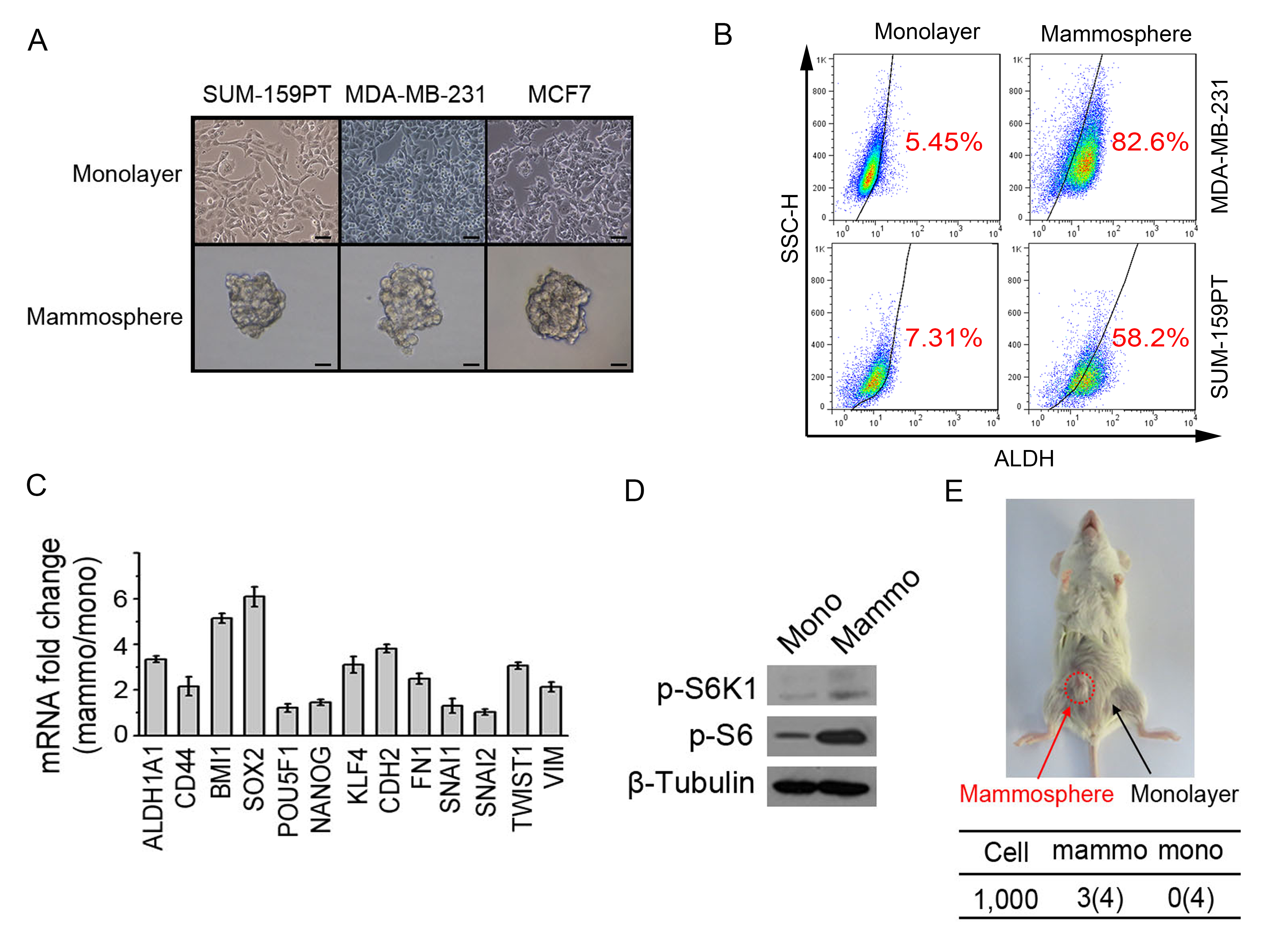
**

**Supplementary Fig. S9. CSCs are enriched in mammospheres.** (A) Representative images of the mammosphere and monolayer cells of SUM-159PT, MDA-MB-231 and MCF7 are shown. Scale bar represents 20 μm. (B) Mammosphere harbors higher ALDH activity. The Aldefluor enzymatic assay of monolayer and mammosphere cells in MDA-MB231 and SUM-159PT cell lines. (C) Expression level of breast CSCs-related markers in SUM-159PT monolayer and mammosphere cells was analyzed by qPCR. The relative fold change was plotted, and data represent mean ± SD from three independent experiments. (D) Phosphorylation of the downstream substrates of PI3K pathway was examined by western blot using lysate from SUM-159PT monolayer and mammosphere cells. (E) Tumor-formation ability of SUM-159PT monolayer and mammosphere cells was analyzed by injection of 1000 cells into the mammary fat pad of NOD/SCID mice. Representative image of tumor-bearing mouse was shown.

**Supplementary Fig. S10**

**
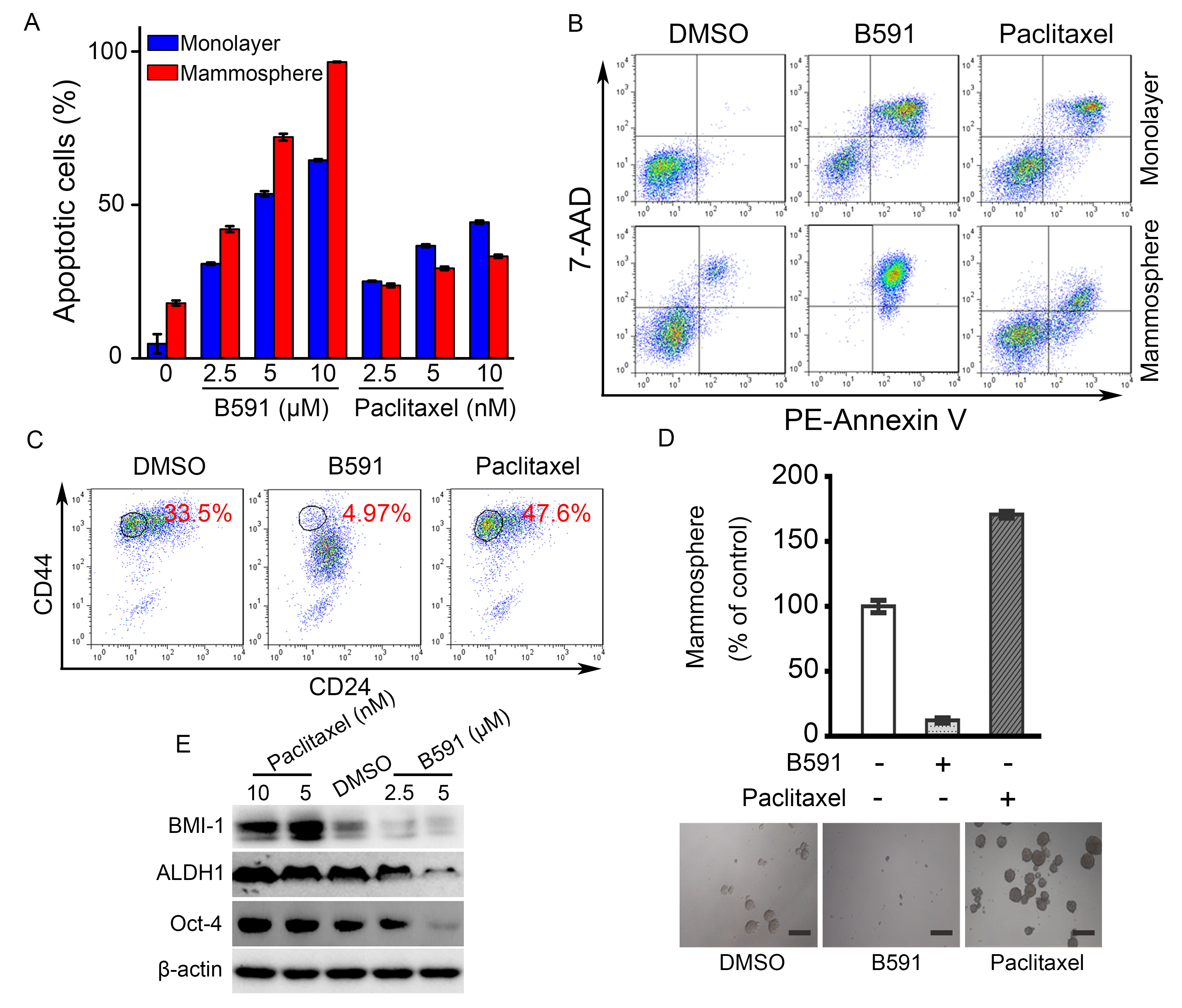
**

**Supplementary Fig. S10. B591 preferentially targets CSCs.** (A) B591 preferentially induced the apoptosis of mammosphere cells of SUM-159PT cells. Cells were treated with indicated agents for 48 h, and cells were harvested and processed for apoptosis assay using the PE-Annexin V/7-AAD Apoptosis Detection Kit. (B) Representative flow cytometry plots of apoptosis inducing activity of B591 (10 μM) and paclitaxel (10 nM) on monolayer and mammosphere of SUM-159PT cells. (C) B591 preferentially targets CD44^high^/CD24^low^ cells. SUM-159PT cells were treated with B591 or paclitaxel for 2 days. Following a 4-day recovery period, cells were co-stained with CD44 and CD24 antibodies and subjected to FACS analysis. Representative flow cytometry plots show the effect of B591 on CD44^high^/CD24^low^ population in SUM-159PT cells. (D) B591 decreased mammosphere forming efficiency. Primary SUM-159PT mammospheres were treated with B591 or paclitaxel for 48 h. Results of the secondary mammosphere-formation assay are shown. Scale bar represents 40 μm. (E) B591 decreased the expression of CSC markers in SUM-159PT cells. Cells were treated with B591 or paclitaxel for 2 days. Following a 4-day recovery period, the expression of CSC markers was analyzed with western blot analysis.

**Supplementary Table S1.** Biochemical kinase profiling data of B591 against 39 human kinases (Life Technologies).

| **Kinase** | **IC_50_ (μmol/L)** | **Kinase** | **IC_50_ (μmol/L)** |
| --- | --- | --- | --- |
| AKT1 (PKB alpha) | >500 | PRKCQ (PKC theta) | >500 |
| AKT2 (PKB beta) | >500 | PRKCZ (PKC zeta) | >500 |
| AKT3 (PKB gamma) | >500 | RPS6KB1 (p70S6K) | >500 |
| AMPK A1/B1/G1 | >500 | SRC | >500 |
| AMPK A2/B1/G1 | >500 | EGFR (ErbB1) | >500 |
| DNA-PK | 448 | ERBB2 (HER2) | >500 |
| FRAP1 (mTOR) | >500 | FLT1 (VEGFR1) | >500 |
| IGF1R | >500 | KDR (VEGFR2) | >500 |
| PDK1 | >500 | FGFR1 | >500 |
| PIK3C2A (PI3K-C2 alpha) | 177 | FGFR2 | >500 |
| PIK3C2B (PI3K-C2 beta) | 91 | FGFR3 | >500 |
| PIK3C3 (hVPS34) | 102 | FGFR4 | >500 |
| JAK1 | >500 | MET (cMet) | >500 |
| PTK2 (FAK) | >500 | PDGFRA (PDGFR alpha) | >500 |
| SYK | >500 | PDGFRB (PDGFR beta) | >500 |
| LYN A | >500 | EPHA1 | >500 |
| LYN B | >500 | EPHA2 | >500 |
| GSK3A (GSK3 alpha) | >500 | EPHA8 | >500 |
| GSK3B (GSK3 beta) | >500 | EPHB1 | >500 |
| FYN | >500 |  |  |

**Supplementary Table S2a.**

| **MW g/mol** | **miLogP** | **TPSA（Å²)** | **N.HBAs** | **N.HBDs** | **nrotb** |
| --- | --- | --- | --- | --- | --- |
| 528.28 | 2.80 | 18.05 | 1 | 0 | 4 |

**Supplementary Table S1a.** Physicochemical parameters of B591 estimated by Molinspiration server. Broadly used Lipinski rule of five (RO5) strategies was implemented by Molinspiration server to quickly understand the physicochemical property of B591 [3, 4]. The physicochemical features of B591 uncovered that B591 obeys the RO5 values except that the molecular weight is slightly greater than 500 Da.

| **Supplementary Table S2b.** | | | | | | | | | | |
| --- | --- | --- | --- | --- | --- | --- | --- | --- | --- | --- |
| **Pharmacokinetics** | | | | | | | | | **Bioavailability** | **Medicinal chemistry** |
| **GI absorption** | **BBB permeant** | **Pgp substrate** | **CYP1A2** | **CYP2C19** | **CYP2C9** | **CYP2D6** | **CYP3A4** | **Log Kp**  **(skin permeation)**  **cm/s** | **score** | **Synthetic accessibility** |
| High | Yes | Yes | No | No | No | No | No | -4.44 | 0.17 | 3.05 |

GI absorption: Gastro intestinal absorption; BBB permeant: Blood‑brain barrier permeability, Pgp‑substrate: P‑glycoprotein‑substrate

**Supplementary Table S1b.** ADME, bioavailability and synthetic accessibility properties of B591. The ADME properties of B591 were calculated using SwissADME server. B591 possesses a high gastrointestinal absorption. The theoretical inhibitory activity of B591 against five cytochrome P450 isoforms (CYP1A2, CYP2C9, CYP2C19, CYP2D6, and CYP3A4) indicates that B591 is not inhibitor to any of the Cytochrome P450 isoenzymes. Besides, the high synthetic accessibility provides the probability in the development of B591.

**Supplementary Table S3.** Sequences of qPCR primers.

| ALDH1A1 forward | ACAAGATCCAGGGCCGTACA |
| --- | --- |
| ALDH1A1 reverse | AGTGCAGGCCCTATCTTCCA |
| GAPDH forward | ACGACCACTTTGTCAAGCTCA |
| GAPDH reverse | TCTCTCTTCCTCTTGTGCTCT |
| CD44 forward | GCAGCCTCAGCTCATACCAG |
| CD44 reverse | TGACTGGAGTCCATATCCATCCTT |
| SOX2 forward | CTGCACATGAAGGAGCACCC |
| SOX2 reverse | CCCGCTCGCCATGCTATTG |
| CDH2 forward | CAACGACGGGTTAGTCACCG |
| CDH2 reverse | CGGGTGCTGAATTCCCTTGG |
| FN1 forward | GAGGAAACCTGCTCCAGTGC |
| FN1 reverse | CACGAACATCGGTGAAGGGG |
| SNAI1 forward | ATGCACATCCGAAGCCACAC |
| SNAI1 reverse | TGCAGTGGGGACAGGAGAAG |
| SNAI2 forward | CGGACCCACACATTACCTTGT |
| SNAI2 reverse | AAAAAGGCTTCTCCCCCGTG |
| KLF4 forward | GGCCCAATTACCCATCCTTCC |
| KLF4 reverse | GGTGGCATGAGCTCTTGGTAAT |
| VIM forward | CAACCTGGCCGAGGACATCA |
| VIM reverse | CGTGCCAGAGACGCATTGTC |
| NANOG forward | AACCTCAGCTACAAACAGGTGAA |
| NANOG reverse | AAAGGCTGGGGTAGGTAGGTG |
| POU5F1 forward | TGGGCTCGAGAAGGATGTGG |
| POU5F1 reverse | ACTGGTCCCCCTGAGAAAGG |
| BMI1 forward | TTGTCTTTTCCGCCCGCTT |
| BMI1 reverse | GTACCCTCCACAAAGCACACA |
| TWIST1 forward | GACCTAGATGTCATTGTTTCCAGA |
| TWIST1 reverse | CCCACGCCCTGTTTCTTTGA |

**References:**

1. Zhou H, Shen T, Luo Y, Liu L, Chen W, Xu B, et al. The antitumor activity of the fungicide ciclopirox. *Int J Cance* 2010;**127**:2467-77.

2. Yu C, Gong Y, Zhou H, Wang M, Kong L, Liu J, et al. Star-PAP, a poly(A) polymerase, functions as a tumor suppressor in an orthotopic human breast cancer model. *Cell Death Dis* 2017;**8**:e2582.

3. Lipinski CA, Lombardo F, Dominy BW, Feeney PJ. Experimental and computational approaches to estimate solubility and permeability in drug discovery and development settings. *Adv Drug Deliv Rev* 2001;**46**:3-26.

4. Shcherbinin DS, Gnedenko OV, Khmeleva SA, Usanov SA, Gilep AA, Yantsevich AV, et al. Computer-aided design of aptamers for cytochrome p450. *J Struct Biol* 2015;**191**:112-9.
